# Differential Localization of the Two *T. brucei* Poly(A) Binding Proteins to the Nucleus and RNP Granules Suggests Binding to Distinct mRNA Pools

**DOI:** 10.1371/journal.pone.0054004

**Published:** 2013-01-30

**Authors:** Susanne Kramer, Bridget Bannerman-Chukualim, Louise Ellis, Elizabeth A. Boulden, Steve Kelly, Mark C. Field, Mark Carrington

**Affiliations:** 1 Department of Biochemistry, University of Cambridge, Cambridge, United Kingdom; 2 Department of Pathology, University of Cambridge, Cambridge, United Kingdom; 3 Department of Plant Sciences, University of Oxford, and Oxford Centre for Integrative Systems Biology, Department of Biochemistry, University of Oxford, Oxford, United Kingdom; The John Curtin School of Medical Research, Australia

## Abstract

The number of paralogs of proteins involved in translation initiation is larger in trypanosomes than in yeasts or many metazoan and includes two poly(A) binding proteins, PABP1 and PABP2, and four eIF4E variants. In many cases, the paralogs are individually essential and are thus unlikely to have redundant functions although, as yet, distinct functions of different isoforms have not been determined. Here, trypanosome PABP1 and PABP2 have been further characterised. PABP1 and PABP2 diverged subsequent to the differentiation of the Kinetoplastae lineage, supporting the existence of specific aspects of translation initiation regulation. PABP1 and PABP2 exhibit major differences in intracellular localization and distribution on polysome fractionation under various conditions that interfere with mRNA metabolism. Most striking are differences in localization to the four known types of inducible RNP granules. Moreover, only PABP2 but not PABP1 can accumulate in the nucleus. Taken together, these observations indicate that PABP1 and PABP2 likely associate with distinct populations of mRNAs. The differences in localization to inducible RNP granules also apply to paralogs of components of the eIF4F complex: eIF4E1 showed similar localization pattern to PABP2, whereas the localisation of eIF4E4 and eIF4G3 resembled that of PABP1. The grouping of translation initiation as either colocalizing with PABP1 or with PABP2 can be used to complement interaction studies to further define the translation initiation complexes in kinetoplastids.

## Introduction

The regulation of translation initiation is one of the most important mechanisms controlling eukaryotic gene expression [1]. In eukaryotes, cap-dependent translation begins with the assembly of the eIF4F complex and eIF4B on the cap at the 5′ end of an mRNA [2]. The eIF4F complex consists of the scaffolding protein eIF4G that binds both the mRNA cap binding protein eIF4E and the RNA helicase eIF4A. The eIF4F complex is involved in the removal of secondary structure from the mRNA 5′ UTR as well as in the recruitment of several further translation initiation factors and the small ribosomal subunit. Scanning of the 5′ UTR results in the recognition of the initiation codon, recruitment of the large ribosomal subunit and release of initiation factors. The efficiency/frequency of translational initiation is increased by closed loop conformation achieved through binding of the poly(A) binding protein (PABP) to both the 3′ poly(A) tail of the mRNA and to translation initiation factors, usually eIF4G. Binding of PABP and eIF4G also stabilizes the interaction between the mRNA cap and eIF4E and has a less well-characterized role in the joining of the small ribosomal subunit.

The interactions described above provide a template that interacts with control processes and many proteins involved in translation initiation also possess regulatory functions. For example, PABPs are involved in both general mRNA metabolism, such as control of mRNA half-life and nonsense mediated decay (NMD) [3], [4], and in mRNA specific functions [5]. The latter includes the regulation of translation by binding to A-rich stretches in 5′ UTRs [6] or 3′ UTRs [7], [8], [9], including the 5′ UTR of its own mRNAs [10], [11], [12], [13], an involvement in miRNA induced silencing by binding to the miRISC complex [14], [15] and contributing to nuclear mRNA processing [16]. Similarly, eIF4E proteins can repress translation of individual mRNAs. This is frequently mediated by eIF4E binding proteins (4E-BPs) that recognize elements in the mRNA 3′ UTR [17]. Complexity is further increased by multiple paralogs of most translation initiation factors that are frequently non-redundant, although the precise functions of these paralogs remain poorly understood. For example, *Arabidopsis* has eight PABP isoforms [18] which differ in domain structure and tissue and/or development specific expression patterns [18], [19], [20], [21], [22]; at least two are independently essential [23]. *Xenopus laevis* has three PABP paralogs: PABP1, ePABP and PABP4, all three isoforms are independently essential with the distinct functions likely being mediated by less-well characterized mRNA specific functions rather than their core role in promoting global translation [24]. Multiple eIF4E paralogs are present in many organisms [17] and it has been suggested that one acts as the translation initiation factor for bulk mRNAs, while the others have regulatory functions both inside and outside of translation [25]. eIF4E paralogs differ in binding affinities for different mRNA cap structures, eIF4G and 4E-BPs. In addition, proteins involved in translation initiation are themselves regulated; for instance, 4E-BP binding to eIF4E is prevented by 4E-BP hyperphosphorylation [26], [27]. Thus, the translational fate of individual mRNAs is determined as a complex function of *cis*-acting elements in both 5′ and 3′ UTRs as well as the availability and activity of different translation initiation factors and regulatory translation factor binding proteins.

The regulation described above is integrated into spatial regulation within the cytoplasm: simplistically mRNAs and translation initiation factors cycle between polysomes and a range of different ribonucleoprotein particles (RNP granules). Both translation initiation factors and poly(A) binding proteins are found in some types of RNP granules. Different types of RNP granules can be present in the same cell and even though the functions of most RNP granules still remain rather elusive, it is likely that the type of RNP granule an mRNA is targeted to will determine its further fate, such as mRNA decay, storage or transport. The best studied are P-bodies, stress granules, neuronal granules and germ granules [28]. P-bodies are constitutively present but change in size and number in response to conditions that affect the translational state of the cell [29], [30], [31]. They contain proteins involved in 5′ to 3′ exonucleolytic mRNA degradation (deadenylases, decapping enzyme DCP1/DCP2, 5′ to 3′ exonuclease), translation factors, components of the nonsense mediated decay (NMD) pathway, miRNA-associated factors, and various mRNA binding proteins [32]. The function of P-bodies is not certain, some data point towards a function in mRNA decay [30], [31], [33] and some do not [34], [35], [36], [37], [38], [39]. Stress granules in mammalian cells differ from P-bodies by the presence of the small ribosomal subunit and absence of components of the mRNA degradation machinery [28]. They are not constitutively present but induced by stress and usually larger and of more irregular shape than P-bodies. However, it is becoming more and more apparent that many different types of stress granules exist and not all fit the criteria originally defined in the mammalian system. In fact, many stresses also cause an increase in size and/or number of P-bodies and the separation between P-bodies and stress granules is not always very clear. For example, the type of stress decides whether budding yeast stress granules contain the 40S ribosomal subunit or not [40], [41], [42], [43] and glucose starvation in fission yeast causes the simultaneous formation of at least two different kind of stress granules that differ in composition [44]. Importantly, P-bodies and stress granules interact with each other [30], [45] and stress granules might even require P-bodies for nucleation [41], [46]. As with P-bodies, the function of stress granules remains largely unknown; they have been suggested to function as storage and sorting place for translationally suppressed mRNAs during stress.

The multiplicity of translation initiation factors and poly(A) binding protein paralogs is not restricted to multicellular eukaryotes. Kinetoplastid protozoa including trypanosomes and Leishmania also have a repertoire: there are two poly(A) binding protein paralogs in trypanosomes (PABP1 and PABP2) and three in *Leishmania* (PABP1, 2 and 3), while both lineages have four eIF4Es, five eIF4Gs and one eIF4A. The reasons for this complexity are unknown, but it suggests a complexity in translation initiation that may have arisen to provide additional levels of regulation in the absence of significant promoter-based transcriptional control in trypanosomes [47].

The amino acid identities between the kinetoplastid PABP paralogs are low, for example 32–33% for the three *Leishmania* isoforms [48]. RNAi depletion of either *T. brucei* PABP paralogs causes growth arrest, indicating that each is independently essential [48]. There is no evidence for life-cycle stage specific expression of PABP paralogs in the experimentally accessible life-cycle stages [49], [50]. In *Leishmania* promastigotes, all three proteins are expressed in excess of mRNA molecules [48]. Poly(A) binding activity has been demonstrated for all three *Leishmania* PABP isoforms [48], [49], [51] and TbPABP2 [52]; PABP2 may have slightly lower affinity towards poly(A) than PABP1 [48]. There is also some evidence for an additional, more regulatory, function for PABP2: (i) PABP2 binds to the CAUAGAAG cycling element present in mRNAs of *Crithidia fasiculata* that show differences in expression levels during the cell cycle; this binding can be competed with poly(A), but not poly(U, G, C) [53]. (ii) PABP2 binds to the U-rich RNA binding protein UBP1 [54], an instability factor of the *T. cruzi* SMUG mucin mRNA [55]. In Leishmania, PABP1 and PABP2 do not co-precipitate, whereas PABP2 and PABP3 do [48], suggesting that PABP1 and PABP2 do not concurrently bind to the same mRNA molecules. The interactions between the poly(A) binding proteins and the eIF4F complex are ambiguous. One report found *in vitro* interactions between all PABPs and eIF4G3 [48], the only eIF4G that was so far shown to interact with eIF4A [56], and *in vivo* interactions between PABP1 and eIF4G3. In contrast, yeast two hybrid analysis failed to detect interactions between PABP1 and eIF4G3, but found PABP1 to directly interact with eIF4E4 instead [57].

In trypanosomes, eIF4E1 and eIF4E2 are of similar molecular weight to their metazoan orthologues and localize to both the nucleus and the cytoplasm. eIF4E3 and eIFE4 have N-terminal extensions and localize to the cytoplasm but not the nucleus [58], [59]. None of the eIF4Es can complement an eIF4E deficient yeast strain [60]. RNAi experiments have shown that at least three of the trypanosome eIF4Es (1, 3, 4) are essential [59]. Interactions between eIF4E4 and eIF4G3 [57], [59], [61], and eIF4G3 and eIF4A [56], [57] have been demonstrated.

Four different types of RNP granules have been described in trypanosomes: P-body-like granules, carbon-source starvation stress granules, heat shock stress granules and nuclear periphery granules (NPGs). Some might be unique to kinetoplastids whereas others have similarities to RNP granules described in mammals and yeast. P-body-like structures [58], [62] show the expected increase when polysomes are dissociated and contain similar components to yeast or mammalian P-bodies. However, no orthologues to the decapping enzymes (DCP1, DCP2, EDC3) have yet been identified in trypanosomes. The cytoplasmic Lsm1-7 complex, a core component of P-bodies in other eukaryotes, is also absent. Instead, SCD6 is essential for P-body formation [63]. Nutrient stress, including naturally occurring restriction in *T. cruzi* epimastigotes, causes formation of starvation stress granules [64]; these granules resemble P-bodies in shape and cytoplasmic localization, but are larger in size and number and contain additional proteins and adenylated mRNAs. On recovery from stress, mRNAs are released from stress granules indicating a function in mRNA storage [64]. Heat shock stress granules are induced by heat shock mediated translational exit and granule formation is accompanied by increased P-bodies and mRNA decay [58]. Nuclear periphery granules have some resemblance to germ granules of animals [63], and form in response to inhibition of *trans*-splicing [63].

Here, the degree of functional redundancy between the two *T. brucei* PABP homologues has been investigated by determining the localisation responses to external stimuli that alter mRNA levels and/or translation. A phylogenetic analysis showed that PABP paralogs have arisen separately many times during the divergence of eukaryotes. Thus, functional differences between trypanosome PABP1 and 2 are unlikely to be generally applicable outside the Kinetoplastae. In trypanosomes, PABP1 and PABP2 localized to different sets of RNP granules in response to inhibition of either translation or *trans*-splicing. PABP2 co-localized with the P-body marker DHH1 into RNP granules with similarity to P-bodies, such as nuclear periphery granules and nutrient starvation stress granules, whereas PABP1 localized to heat shock induced stress granules. On puromycin treatment, PABP1 and 2 fractionated differently on a sucrose gradient, suggesting, that the two proteins may assemble to distinct RNPs at translational exit. Interestingly, the behaviour of the PABPs was mirrored by the different eIF4F isoforms: eIF4E1 had similar localization behaviour to PABP2, whereas eIF4E4 and eIF4G3 localization resembled that of PABP1. We propose that localization studies to RNP granules can be used complementary to binding studies to unravel the still poorly defined translation complexes in trypanosomes.

## Results

### Paralog Expansion of PABPs has Occurred Independently Several Times in Eukaryotic Evolution and Prior to Kinetoplastid Divergence

**Figure 1:**
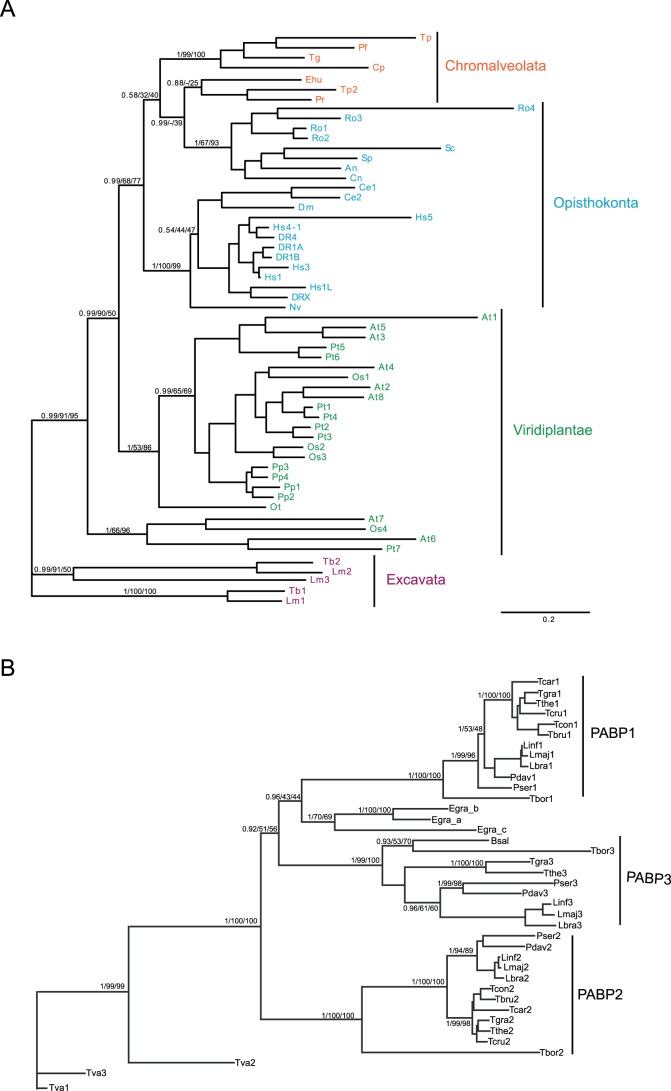
Duplication and divergence of PABPs has occurred on multiple occasions during eukaryote evolution. A) Phylogenetic reconstruction of PABP evolutionary history using predicted protein sequences from four eukaryotic supergroups [78]. Phylogenetic analysis was performed with MrBayes, RAxML and PhyML locally, using the WAG model for ML and mixed parameters for MrBayes [79], [80], [81]. The tree was generated using the MrBayes topology. Numbers indicate Baysian posterior probabilities and bootstrap support for PhyML and RAxML respectively; a dash indicates a feature not reconstructed under a specific algorithm. Leaves are colorized according to supergroup: green for Viridiplantae, light blue for Opistokhonta, orange for Chromalveolates and purple for Excavata. Taxa are indicated by a two letter code based on Linnean nomenclature; Hs; *Homo sapiens*, Dm; *Drosophila melanogaster*, Ce; *Caenorhabditis elegans*, DR; *Danio rerio*, Nv; *Nematostella vectensis*, Cn; *Cryptococcus neoformans*, Sp; *Schizosaccharomyces pombe*, An; *Aspergillus nidulans*, Ro; *Rhizopus oryzae*, At; *Arabidopsis thaliana*, Ot; *Oryza sativa*, Pt; *Populus trichocarpa*, Pp; *Physcomitrella patens*, Ot; *Ostreococcus tauri*, Pf; *Plasmodium falciparum*, Tg; *Toxoplasma gondii*, Cp; *Cryptosporidium parvum*, Tp; *Theileria parva*, Pr; *Phytophthora ramorum*, Tb; *Trypanosoma brucei* and Lm; *Leishmania major*. Note that he apparent split of the Viridiplantae clade is an artefact of the manner in which the tree has been drawn, and that monophyly is well supported. **B)** Phylogenetic reconstruction of PABPs within the Excavata prepared as above. Taxa are indicated by a three letter code based on Linnean nomenclature: Tva, *Trichomonas vaginalis*; Egr, *Euglena gracilis*; Tbo, *Trypanoplasma borrelli*; Bsa, *Bodo saltans*; Pse, *Phytomonas serpens*; Pda, *Phytomomas davidii*; Lb, *Leishmania braziliensis*; Lin, *Leishmania infantum*; Lma, *Leishmania major*; Tbr, *Trypanosoma brucei*; Tco, *Trypanosoma congolense*; Tcr, *Trypanosoma cruzi*; Tth, *Trypanosoma theileri*; Tgr, *Trypanosoma grayi* and Tca, *Trypanosoma carassii*.

To investigate whether the PABP paralogs in divergent species are likely to be orthologous, a phylogenetic analysis was performed to determine whether expansion of paralogs occurred before or after the divergence of different species. Predicted PABP protein sequences were identified on the basis of sequence identity, the presence of four RRM domains (pfam00076) and the conserved and unique PABP C-terminal domain (pfam00658). A phylogenetic analysis of PABPs from representative organisms from four of five recognised eukaryotic supergroups is shown in Figure 1a (see Table S1 for the sequences). Evidence for paralogous expansion is clear in all clades, with the exception of the Chromalveolates that appear to have a single representative in each organism. Further, the Opistokonta clade was not resolved as monophyletic, with a clear division between the metazoa and the fungal lineages; this may reflect rapid selective pressure, particularly in the fungi where several branches are rather long. Additionally, there was evidence for high rates of divergence amongst plants. Regardless, in all cases the paralogs were reconstructed as lineage-specific, suggesting that the expansions of PABPs occurred after the initial differentiation of the supergroups and post the last eukaryotic common ancestor. This suggests that, while selective pressure for duplication of PABPs and hence need for functional diversification is common across the eukaryotic lineage, these are independent events in evolutionary history, with the functional consequence that division of labour between the resultant paralogs is likely specific to each supergroup.

Trypanosomes have two PABP paralogs whereas Leishmania has three: PABP1, 2 and 3. To investigate the origin of this difference, a phylogenetic analysis of PABPs from thirteen diverse Kinetoplastea including eleven Kinetoplastids and two Bodonids. *Euglena gracilis* and *Trichomonas vaginalis* were included as more divergent Excavates. The phylogenetic analysis (Figure 1b) supports a model in which the last common ancestor to the Kinetoplastae contained three paralogs, still extant in *Trypanoplasma borrelli*, *Leishmania* sp. and *Phytomonas* sp. The last common ancestor of all *Trypanosoma* sp. (Kinetoplastae) had lost PABP3 whereas *Bodo saltans* (Bodonida) has lost PABP1 and 2. The more distantly related Excavates, *E. gracilis* and *T. vaginalis* have undergone separate expansion of paralogs. These findings seem to suggest some evolutionary redundancy between PABP paralogs in Kinetoplastae in that no single ortholog is conserved in all species.

### Localisation Suggests a Functional Difference between PABP1 and PABP2

In contrast to the apparent evolutionary redundancy, it has been shown by RNAi knock-down that PABP1 and PABP2 are independently necessary for cell proliferation in *Trypanosoma brucei* [48]. This finding was confirmed with independent constructs (Figure S1). Thus, in trypanosomes PABP1 and 2 are not functionally redundant and in experiments investigating the subcellular localisation of PABP1 and PABP2 differences were observed and then investigated more systematically by determining changes in the localisation of PABP1 and 2 in response to stress.

Trypanosomes are diploid and various procyclic form cell lines were made that contained modified versions of one allele at each of the PABP1 and PABP2 loci so that the cells expressed one, the other, or both PABP1-eYFP and PABP2-mChFP/eYFP fusion proteins [63]. The PABP1-eYFP protein is functional for procyclic form growth as deletion of the second allele results in only a minor growth effect (Figure S2), whereas RNAi depletion of PABP1 is lethal (Figure S1). It is possible that the tagged PABP2 is not fully functional as attempts to delete the second allele were not successful. However, all localizations of tagged PABP2 described below were identical to localizations obtained by immunofluorescence using an antiserum raised against Leishmania major PABP2 (Figure S3), indicating that the fluorescent protein tag did not affect the localization.

**Figure 2:**
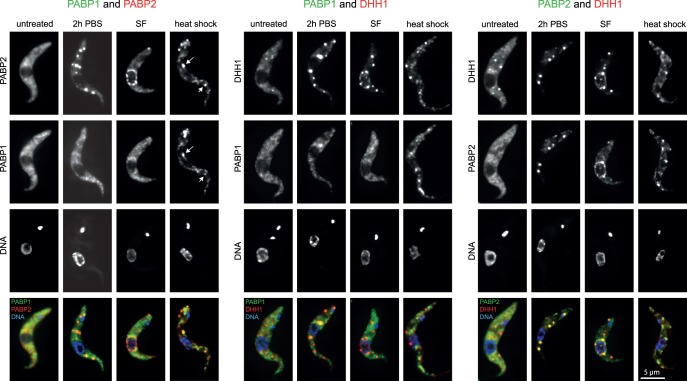
Differential localization of PABP1 and PABP2 to inducible RNP granules. Z-stack projections of fluorescent microscopy images of cells coexpressing PABP1-eYFP and PAPB2-mChFP, PABP1-eYFP and mChFP-DHH1 or PABP2-eYFP and mChFP-DHH1 are shown. Cells were either untreated or treated with nutrient starvation (2 h PBS), heat shock or sinefungin. The arrows point to heat shock induced PABP1 and PABP2 granules that colocalize. Note that for the heat shock experiment of the cell line expressing PABP1 and DHH1 fusions a cell line was used expressing PABP1-mChFP and eYFP-DHH1; images are shown in false colors for clarity.

To investigate any relationship between PABPs and P-bodies, cells lines were made that expressed the P-body marker mChFP-DHH1 [58] and either PABP1-eYFP or PABP2-eYFP. The localization of the fluorescently tagged proteins was determined in response to nutrient starvation (2 hours PBS), heat shock (2 hours at 41°C) and inhibition of *trans*-splicing (1 hour sinefungin at 2 µg/ml) (Figure 2).

As previously reported, both PABPs were localised in the cytoplasm in the absence of stress and did not localize into microscopically visible RNP granules. However, localization in response to stresses differed. In response to nutrient starvation, the majority of PABP2 localized to starvation granules co-localizing with DHH1, while PABP1 was largely, although not entirely, absent from these granules. In response to heat shock, both proteins localized to granules some of which colocalized (arrows in Figure 2 and Figure S4A–C). In general, PABP2 did largely but not entirely colocalize with the P-body marker DHH1 on heat shock treatment, while PABP1 largely did not. As previously reported, PABP2 co-localized with DHH1 in sinefungin induced nuclear periphery granules as well as into sinefungin induced cytoplasmic P-bodies, while PABP1 was completely absent from either type of granules (Figure 2 and [63]).

Experiments were repeated using procyclic form cell lines containing tetracycline-inducible transgenes for either PABP1-eYFP or PABP2-eYFP under the control of a strong promoter. After 24 h induction, expression of PABP2-eYFP increased about five fold when compared with expression from the endogenous locus. There was a slight decrease in the expression of the endogenous PABP2 (Figure S5A), possibly by the feedback regulation of poly(A) binding proteins by A-rich stretches in their 5′ UTR [13], a mechanism that may be conserved for *T. brucei* PABP2 [65]. After induction, PABP1-eYFP expression levels were between 2 and 5 fold higher than expression levels from the endogenous locus. The localization of the over-expressed PABPs was unaltered, with the one exception that a larger fraction of PABP1 localized to starvation stress granules, although still less than found for PABP2 (Figure S5B). One possible explanation is that PABP1 is actively prevented from localization to starvation stress granules by factors that become limiting in response to overexpression of PABP1. The localization of PABP1 to starvation stress granules in response to overexpression can explain the discrepancy to a study done in *T. cruzi* that reports both PABPs to be present in starvation stress granules when expressing eYFP fusions [64].

There are major differences between PABP1 and PABP2 in localization to RNP granules, with PABP2 localizing exclusively to RNP granules with some relationship to P-bodies, such as cytoplasmic P-bodies induced by either heat shock or sinefungin, starvation stress granules or nuclear periphery granules, while PABP1 is largely absent from these granules types and localizes to heat shock stress granules in response to heat shock. These differences remain, when the proteins are overexpressed, with the exception that the amount of PABP1 protein present in starvation stress granules increases. The data suggest that the two poly(A) binding proteins may interact with different sets of mRNAs destined for distinct fates during conditions resulting in RNP granule induction/translational repression.

### PABP1 and PABP2 Fractionate Differently on Sucrose Gradients on Puromycin-induced Polysome Dissociation

**Figure 3:**
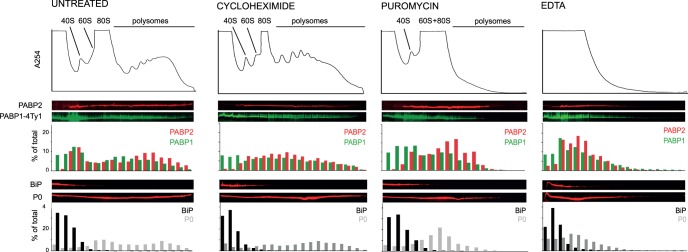
Distribution of TbPABP1 and TbPABP2 across sucrose gradients. Lysates prepared from a cell line expressing PABP1-4Ty1 treated as indicated were fractionated by sucrose gradient centrifugation. PABP1-4Ty1 and PABP2, as well as the control proteins BiP and P0 were detected across the fractions of the gradients by western blotting. The absorption profile of the sucrose gradient at 254 nm, the western blots and western blot quantifications are shown.

Sucrose density gradient centrifugation was used to separate polysomes from cytoplasm and the subsequent distribution of PABP1 and PABP2 was examined. The cell line used for these experiments carried a transgene encoding PABP1-4TY1 (four tandem Ty1 epitope tags at the C-terminus [66]) to allow the detection of both PABP1 and 2 on the same western blot. The transgene was made by modification of the endogenous locus. Two further protein markers were used to analyse the distribution of cellular contents: P0, a large ribosomal subunit protein was used to detect 60S subunit, monosomes and polysomes; BiP served as a control for non-polysome associated proteins. Lysates were prepared from: (i) untreated cells, (ii) cells pre-incubated for 30 minutes with either cycloheximide or anisomycin, both elongation inhibitors that cause ribosomes to arrest on the mRNA; (iii) cells pre-incubated for 30 minutes with puromycin resulting in premature chain termination and release of ribosomes from mRNAs. When used, inhibitors were also present during preparation of the lysate. Each analysis was performed at least three times in at least two independent experiments (Figure 3, Figure S6). In untreated cells, both PABPs were present in all fractions of the gradient corresponding to mono and polysomes. The vast majority of PABP2 entered the gradient, whereas a significant fraction of the PABP1 did not. About half of PABP2 (57%, 53%) and slightly less of PABP1 (44%, 33%) was present in fractions corresponding to polysomes (Figure 3, Figure S6). The distribution of both proteins across the gradient did not change significantly, when cycloheximide or anisomycin were added: 58±4% of PABP2 and 45±8% of PABP1 was present in fractions corresponding to polysomes (average values from three experiments +/− standard deviation) (Figure 3, Figure S6). Significant differences in distribution were found when polysomes were dissociated by puromycin treatment. The majority of PABP1 did not enter the sucrose gradient with only some present in fractions corresponding to monosomes. In contrast, the majority of PABP2 was present in fractions corresponding to monosomes and complexes larger than monosomes (Figure 3 and Figure S6). 41 and 33% of PABP2 was still present in fractions corresponding to the remaining polysomes in the two quantitated experiments, but only 17 and 7% of PABP1. EDTA disrupts polysomes and possibly RNP complexes and after EDTA addition both proteins were found in identical fractions at the top of the sucrose gradient, equivalent to the distribution of the P0 protein.

The interpretation of these data is non-trivial, as the method cannot distinguish between association with polysomes/monosomes, and association with other RNP complexes. However, the absence of both poly(A) binding proteins from high-density sucrose fractions after disruption of polysomes strongly indicates that at least some of each protein is truly associated with polysomes. The differences observed after dissociation of polysomes suggest that PABP1 and PABP2 localize to different RNP complexes when not present in a polysome: consistent with the localization to different types of RNP granules described above.

As previously reported [48], [49], we also noticed that PABP1, but not PABP2, was detectable in multiple forms on a western blot (Figure 3 and Figure S6), presumably representing different post-translationally modified forms. Some phosphorylations of ePABP from *Xenopus* have recently been shown to be required for polysomal association [67], while methylations of mammalian PABP1 are not required for polysomal association [68]. In trypanosomes, no significant changes in the PABP1 band pattern were observed between the different sucrose fractions, indicating that the modifications are not specifically associated with polysomal PABP1 or PABP1 in a complex of a certain size. This was further confirmed by examining the PABP1-4TY1 band pattern in cells treated with conditions that disrupt translation (cycloheximide, puromycin and heat shock) or mRNA processing (actinomycin D, sinefungin) (Figure S7). There was no significant change in the PABP1-4Ty1 band pattern in response to inhibition of translation but there was a decrease from five to four bands, when mRNA processing was inhibited, consistent with previous findings [49]. These data support the finding that none of the modifications that are detectable by Western blot are specific for either polysomal or non-polysomal PABP1.

### TbPABP2, but not TbPABP1 Localizes to the Nucleus after Combined Heat Shock and Sinefungin Treatment, but a Predicted NLS, RLRRER, is Neither Necessary Nor Sufficient

**Figure 4:**
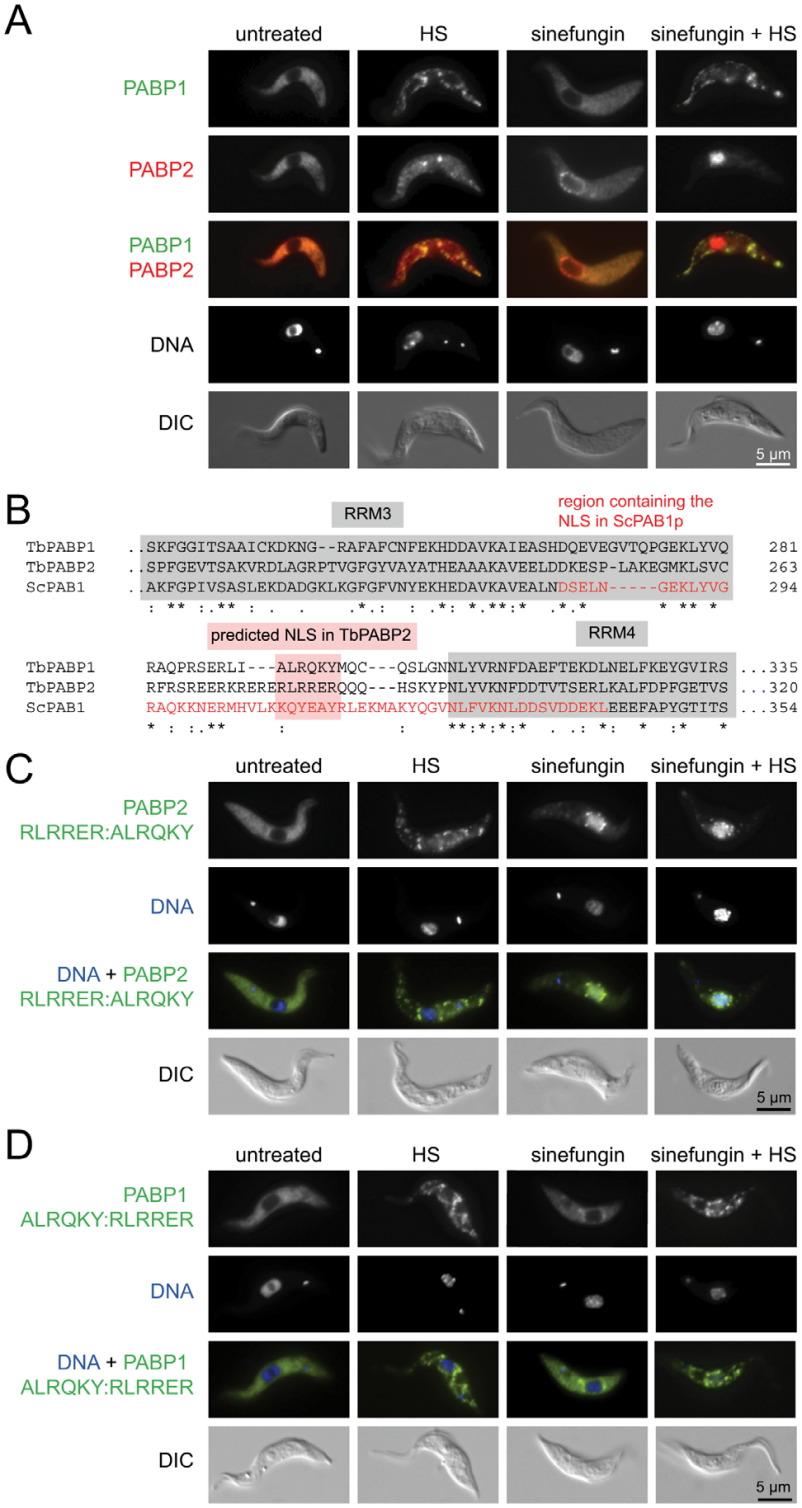
The predicted NLS of PABP2 is not an NLS. A) Fluorescent microscopy images of cells expressing PABP1-eYFP/PABP2-mChFP either untreated or treated with sinefungin (2 µg/ml), heat shock (41°C) or both sinefungin and heat shock for two hours. **B)** Alignment of TbPABP1, TbPABP2 and ScPAB1p (region between RRM 3 and 4). The predicted NLS of TbPABP2 is highlighted, as well as the NLS of the yeast protein PAB1p [74]. **C and D)** The predicted NLS of *T. brucei* PABP2 (RLRRER) was exchanged with the equivalent region from PABP1 (ALRQKY) (**C**) and vice versa (**D**). Both mutant proteins had cytoplasmic localization identical to the wild type proteins (not shown). Fluorescent microscopy images of untreated cells as well as cells treated with sinefungin, heat shock or both are shown.

One further difference in intracellular localization between the PABPs has been previously reported in *Leishmania*, PABP2 accumulated in the nucleus when transcription was inhibited by actinomycin D [48], similar behaviour to human PABP1 [69] whereas there is little accumulation of PABP1 in the nucleus [49]. Nuclear accumulation of PABPs has been shown to correlate with nuclear mRNA accumulation, indicating mRNA dependent nuclear export of PABPs [70]. Using the cell line expressing PABP1-eYFP and PABP2-mChFP, only a minor nuclear localization of PABP2 was found at later time-points of an incubation with actinomycin D (data not shown). However, when mRNA maturation was inhibited using sinefungin, a fraction of PABP2, but not PABP1, was found in the nucleus (Figure 4A). When two hours of sinefungin treatment were combined with heat shock, which inhibits export of mRNA from the nucleus in yeast [71], [72], [73], a large fraction of PABP2 accumulated in the nucleus, whereas PABP1 was in the cytoplasm, mostly in heat shock stress granules (Figure 4A).

These differences in nuclear localization may be due to the presence of a nuclear localisation signal in PABP2, but not in PABP1. PABP2 has a predicted nuclear localization signal, RLRRER, between RRM3 and RRM4 that is absent in PABP1 [48]; the NLS of the *S. cerevisiae* poly(A) binding protein is in the equivalent location [74] (Figure 4B). To test whether this predicted NLS is necessary for heat-shock plus sinefungin mediated localization to the nucleus, the RLRRER sequence of PABP2 was replaced by the corresponding sequence present in PABP1 (ALRQKY) and a cell line was made containing a transgene encoding the mutated PABP2-eYFP under the control of an inducible promoter. The PABP2 RLRRER::ALRQKY-eYFP localized to the nucleus in response to heat-shock and sinefungin treatment (Figure 4C), indicating that the RLRRER sequence is not necessary for nuclear localization of PABP2. To test whether RLRRER is sufficient for nuclear localization, the equivalent PABP1 mutant protein, PABP1 ALRQKY::RLRRER-eYFP was tested for nuclear localization: the majority of the PABP1 mutant was still in the cytoplasm after heat-shock and sinefungin treatment, indicating that the RLRRER sequence is not sufficient for nuclear localization either (Figure 4D). Both PABP mutant proteins showed minor differences in localization with sinefungin treatment in comparison to the wild type. PABP2 RLRRER::ALRQKY-eYFP accumulated to a slightly greater extent in the nucleus and PABP1 ALRQKY::RLRRER-eYFP showed minor localization to NPGs (Figure 4C and D). This was not further examined in this study.

When RLRRER was expressed fused to a double tomato fluorescent protein (dTFP), the fusion protein localized to both the cytoplasm and the nucleus in equal amounts, as did a randomly chosen sequence (GGHESSVE) fused to dTFP. In contrast, the majority of a fusion of dTFP to a previously defined *T. brucei* NLS, RGHKRSRE [75], localized to the nucleus (Figure S8 A and B). Together, the data show that the predicted NLS RLRRER is neither necessary nor sufficient for nuclear import and is not an NLS. There are several arginine residues upstream of the RLRRER sequence and to exclude the possibility that the RLRRER sequence was too short to mediate nuclear import, larger regions were expressed as dTFP fusion proteins (Figure S8 B). While two of these sequences caused a minor accumulation of the fusion protein in the nucleolus, the majority of each fusion protein remained equally distributed throughout the cytoplasm and nucleus, similar to the control, the random sequence (Figure S8 B). To summarize, PABP2, but not PABP1, can localize to the nucleus when cells are treated with sinefungin and heat shock, but the predicted NLS, RLRRER, present in PABP2 is neither necessary nor sufficient for nuclear localization. The NLS of PABP2 probably belongs to the less defined non-classical type of NLS, like seen for other trypanosome RNA binding proteins [76], [77], as well as for yeast and human Poly(A) binding proteins [69], [74].

### Co-localization of Other Translation Initiation Factors with PABP1 and 2 to Inducible RNA Granules

**Figure 5:**
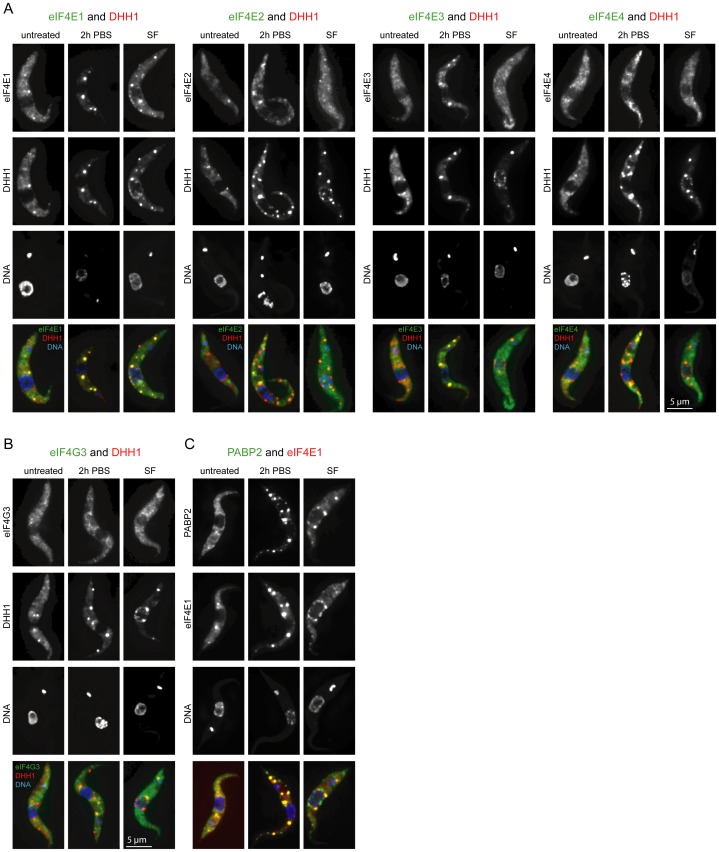
Differential localization of translation initiation factors to inducible RNA granules. **A)** eYFP fusions of eIF4E1-4 with mChFP-DHH1. **B)** eIF4G3-eYFP and mChFP-DHH1. **C)** PABP2-eYFP and eIF4E1-mChFP.

The localization of the four *T. brucei* eIF4E isoforms and eIF4G3, an eIF4G isoform that interacts with eIF4A [56], to nutrient starvation stress granules and sinefungin-induced granules was examined. In all cases, the eYFP-fusion transgenes were made by modification of the endogenous locus in a cell line expressing mChFP-DHH1; eIF4E1-eYFP was also co-expressed together with PABP2-mChFP (Figure 5). As previously reported, only eIF4E1 partially localized to cytoplasmic P-bodies in untreated cells [63] and when granule formation was induced, a large fraction of eIF4E1 co-localized with DHH1 to starvation stress granules and nuclear periphery granules, as well as to sinefungin induced cytoplasmic P-bodies, exhibiting a similar behaviour to PABP2 (Figure 5C). In contrast, eIF4E2 and eIF4E3 showed only minor localization to starvation stress granules and were not detectable in nuclear periphery granules. eIF4E4 and eIF4G3 showed a localisation pattern similar to PABP1, a small fraction was visible in starvation stress granules and none was detectable in nuclear periphery granules. Similar experiments were performed in cell lines expressing eYFP fusions of each of the eIF4E isoforms or eIF4G3 together with PABP1-mChFP, leading to identical results (Figure S9). Thus, eIF4E1 localization to RNP granules resembles that of PABP2, while eIF4E4 and eIF4G3 localization resembles that of PABP1.

## Discussion

There is increasing evidence that the evolutionary divergence of kinetoplastid protozoa is reflected in subtle differences in the mechanism and control of translation initiation. The interactions between the different orthologues of translation initiation factors are still not well understood. This work provides an alternative strategy for establishing which paralogs may be part of a particular pathway by examining the intracellular localization of the different paralogs under conditions of translational repression or inhibition of trans-splicing (summarized in Table 1). We found significant differences between PABP1 and PABP2 in localization to the four different types of RNP granules that can be induced in trypanosomes. In addition, there was a difference in localization to the nucleus under conditions that probably inhibit nuclear export and in their behaviour on sucrose gradients after puromycin induced inhibition of translation. Moreover, the translation factor eIF4E1 localized to both nutrient starvation stress granules and nuclear periphery granules, similar to PABP2, while eIF4E4 and eIF4G3 were absent from either granule type, similar to PABP1. eIF4E2 and eIF4E3 showed an intermediate behaviour: partial localization to nutrient starvation stress granules but absence from nuclear periphery granules and it is possible they represent a third type of localisation behaviour. The differences in localization to inducible RNP granules between P-body markers, eIF4E1 and PABP2 on the one hand and PABP1, eIF4E4 and eIF4G3 on the other hand strongly indicate that the proteins are part of two distinct pathways that interact with distinct sets of mRNAs; the data are consistent with previously published interactions between eIF4E4 and eIF4G3 [57], [59], [61], PABP1 and eIF4E4 [57] as well as with the lack of interaction between PABP1 and PABP2 [48].

**Table 1:** Summary of localization to granules and co-localization shown in this study.

	P-bodies		Heat shock granules	PBS		NPGs	
		coloc. with			coloc. with		coloc. with
**DHH1**	XXX	eIF4E1	n.d.	XXX	PABP2, eIF4E1-3	XXX	PABP2, eIF4E1
**PABP1**	−	−	XXX	−^2*^	−	−	−
**PABP2**	−	−	X (+ PBs)	XXX	DHH1, eIF4E1	XXX	DHH1, eIF4E1
**eIF4E1**	XX	DHH1	n.d.	XXX	PABP2, DHH1	XXX	PABP2, DHH1
**eIF4E2**	−^1*^	−	n.d.	XX	DHH1	−	
**eIF4E3**	−^1*^	−	n.d.	XX	DHH1	−	
**eIF4E4**	−^1*^	−	n.d.	−	−	−	
**eIF4G3**	−^1*^	−	n.d.	−	−	−	

1 *low expression levels.

2 *localization to PBS granules at over-expression.

### What is the Underlying Biological Function Linked to the Localisation?

NPGs are unlikely to contain mRNAs that have been previously in translation, as they form faster than polysomal disruption and are insensitive to cycloheximide [63]. Instead, their sensitivity to actinomycin D suggests that they may contain newly transcribed mRNAs [63]. It is therefore possible that the protein components of the NPGs originate from the nucleus, raising the possibility that the PABP2 containing complex may bind to mRNAs in the nucleus, while the PABP1 containing complex may bind later. This would be consistent with the observation that PABP2 but not PABP1 accumulates in the nucleus at conditions that probably prevent nuclear export.

Heat shock causes the formation of both heat shock stress granules as well as the induction of P-body like granules. While PABP2 is mainly present in the P-body like granules, PABP1 is mainly found in the heat shock stress granules and to a smaller fraction in the P-body like granules. The function of both granule types is entirely unknown. It is tempting to speculate that one may be involved in the heat shock induced rapid decay of mRNAs [58], possibly the P-body like type, as it contain the cytoplasmic 5′-3′ exoribonuclease XRNA, while the other type may be specialized in mRNA storage. This way the binding to a particular isoform of PABP would determine the fate of the mRNA at heat shock induced translational repression.

Nutrient starvation stress granules act most likely as mRNA storage granules as they can be stained with both oligos antisense to poly(A) or the mini-exon, indicating they contain intact mRNAs [63], [64] and mRNAs can return to the cytoplasm at stress recovery [64]. While PABP2 localizes to starvation stress granules at both low and high level of expression, the localization of PABP1 to starvation stress granules was dependent on the expression level. It is possible, that PABP1 is usually prevented from localization to starvation stress granules by binding to a factor that becomes limiting at overexpression.

The differences in localization to RNP granules between the two PABP isoforms are complemented by differences in distribution across sucrose gradients. Generally, PABP2 was present in higher density sucrose fractions than PABP1. While the differences were less obvious in untreated cells or cells that had been treated with polysome stabilizing drugs, they became apparent when polysomes were disrupted by puromycin. It is clear, that the proteins associate with distinct (submicroscopic) RNP complexes when polysomes are dissolved, in agreement to their localization to different RNP granules when translation or trans-splicing is disrupted.

Both trypanosome PABP paralogs examined so far are essential, do not bind to each other and the functional differences are likely to be lineage specific. Differences in intracellular localizations under various conditions that alter mRNA metabolism are striking and predict association with distinct cohorts of mRNAs. Similar differences in localization to RNP granules were observed for translation initiation factors of the eIF4F complex and we propose that studies based on localization of proteins at translational repression can be used complementary to binding studies to gain inside into the mechanisms of regulation of translation in trypanosomes and elsewhere.

## Material and Methods

### Trypanosomes

*Trypanosoma brucei* Lister 427 procyclic cells (a kind gift of K. Gull [82]) were used for all experiments involving expression of proteins from their endogenous loci. RNAi was performed in Lister 427 29-13 cells (Wirtz 1999) and inducible expression in either Lister 427 pSPR2 cells [83] for dTFP fusion proteins; Lister 427 PTT cells (a kind gift of Philippe Bastin [84] REF) or Lister 427 SIMP (a kind gift of Bill Wickstead [85]) for the PABP experiments. Transgenic trypanosomes were generated using standard procedures [86]. All experiments were performed with logarithmically growing trypanosomes at a cell density of less than 1×10^7^ cells/ml.

### Plasmids and Cloning

All plasmids used in this work for endogenous or inducible expression of fusion proteins as well as for RNAi are summarized in Table S2. For deletion of one PABP1 allele, the open reading frame of a PABP1 gene was replaced by puromycin; the plasmid contained 930 nucleotides upstream of the PAPB1 ORF and 1471 nucleotides downstream for homologous recombination (p3743).

### Microscopic Imaging

Cells were washed with SDM79 without serum and fixed at a density of 1*10^7^ cells/ml with 2.4% paraformaldehyde overnight, washed once in PBS and stained with Hoechst H33258. Fluorescence microscopy was carried out using a Zeiss Axioskop microscope equipped with a Plan-Apochromat 100/1.4 Oil DIC objective. Images were taken with the monochrome CCD camera AxioCam MR using AxioVision software (Zeiss). For Figure 2, 5 and S10 Z-stack images (100 stacks at 100 nm distance) were taken with a custom build TILL Photonics iMic microscope equipped with a sensicam camera (PCO), deconvolved using Huygens Essential software and presented as Z-projections (method maximal intensity) produced by ImageJ software. The filter sets 49008 - ET - mCherry, Texas Red and 49003 - ET – EYFP (Chroma Filter Set, Chroma Technology) were used for mChFP and eYFP, respectively; with these filter sets there was no detectable bleed-through.

### Blots

Southern blots and western blots were performed according to standard procedures, northern blots as described before [58]. Detection of proteins was done using the Odyssey Infrared Imaging System (LI-COR). For quantification, the Odyssey software was used (background method: the average of a three pixel width line at the top and bottom of each band was subtracted from each pixel).

### Polysome Gradients and Concentration of Protein Samples

Polysome analysis was performed as previously described [58]. Cycloheximide, anisomycin and puromycin were used at 50 µg/ml, 30 µg/ml (1∶1666 stock in DMSO) and 50 µg/ml, respectively and added to the cells 30 minutes prior to harvesting as well as to the wash buffer. Control cells treated with DMSO (1∶1666) gave similar A254 profiles and protein distribution similar to untreated cells. Cell lysate incubated with EDTA on ice for 30 minutes prior to the loading served as control.

For detection using BB2 monoclonal antibody and anti-PABP2, proteins from each fraction were concentrated 10 fold by adding 600 µl methanol, 150 µl chloroform and 450 µl water to 150 µl of each sucrose fraction, vortexing after each step. Samples were centrifuged (5 min, 13.000 g), the upper aqueous layer was discarded. Proteins were precipitated by mixing with 650 µl methanol and 5 min centrifugation (5 min, 13.000 g). Pellets were allowed to air-dry, dissolved in 10 µl 1× sample buffer and boiled for 5 minutes.

### Kinetoplast PABP Sequences

The sequences used for the phylogenetic tree were obtained from public databases with the exception of the following: *E. gracilis, T. carassii, T. borrelli* and *T. theileri* were derived from recent transcriptome sequencing projects in the MC lab, *T. grayi* from a genome sequencing project in the MCF lab, *Phytomonas* species from a transcriptome sequencing project in the SKe lab and *Bodo saltans* from Andrew Jackson (http://www.sanger.ac.uk/resources/downloads/protozoa/bodo-saltans.html).

## Supporting Information

Figure S1 **RNAi knock-down of either TbPABP1 or TbPABP2 is lethal.** PABP1 **(A)** and PABP2 **(B)** knock-down by tetracycline (TET) inducible RNAi. Both growth (top) and the reduction in PABP1 and PABP2 mRNAs (bottom) were monitored over a time-course of RNAi induction in procyclic cells. RNA from wild type cells (wt) served as control. Loading of the northern blots was controlled by reprobing for ribosomal RNA.
(PDF)

Figure S2 **Verification and analysis of PABP1-eYFP/Δpabp1 procyclic cells. A)** To-scale maps of the PABP1 genomic locus: wild type (top), after replacement of the PABP1 gene by a puromycin resistance gene (ΔPABP1, middle) and after integration of PABP1-eYFP (bottom). AgeI restriction enzyme sites and positions of the probes used for the Southern Blot in B) are indicated. **B)** Verification of the transgenic cell lines by Southern blot. **C)** Growth curves of wild type cells and transgenic cell lines. Population doubling times (PDT) are indicated.
(PDF)

Figure S3 **Detection of PABP2 in inducible RNP granules by immunofluorescence.** 2*10^7^ cells, untreated or stressed by carbon source starvation (3 hours PBS), 2 h heat shock at 41°C (HS), sinefungin (SF) or 2 hours heat shock and sinefungin (HS+SF) were washed once in SDM79 without serum and heme, resuspended in 5 ml SDM79 without serum and heme and fixed for 15 minutes with 1 volume 8% paraformaldehyde in PBS at RT while rotating. Cells were washed once in PBS and allowed to settle on slides for 15 minutes. Slides were washed in 25 mM NH_4_Cl for 10 minutes. Cells were permeabilized and blocked for one hour in blocking solution (taken from fluorescent antibody enhancer set for DIG detection, Roche) containing 0.5% saponin. Slides were washed, blocked for an additional 30 minutes without saponin, incubated with the first antibody (anti-LmPABP2 1:500 or anti-GFP, Invitrogen A11122, 1∶100) for 60 minutes, washed 4 times in PBS, incubated with the secondary antibody (anti-mouse Alexa 568) and mounted in FluorSave (Calbiochem), containing Höchst33342 DNA stain at 5 µg/ml. Wild type cells stained with anti-GFP or cells stained with no antibody served as controls and showed significantly less signal (not shown).
(PDF)

Figure S4 **Localization of PABP1 and PABP2 in response to heat shock (2 hours at 41**°**C). A)** Cells expressing PABP1-eYFP and PABP2-mChFP. **B)** Cells expressing PAPB2-eYFP. **C)** Cells expressing PABP1-eYFP. All fusion proteins were expressed from their endogenous loci.
(PDF)

Figure S5 **Inducible over-expression of PABP1-eYFP and PABP2-eYFP. A)** Western blots: 5*10^6^ cell equivalents of cells induced to over-express PABP1-eYFP or PABP2-eYFP for 24 or 48 hours (TET) or cells expressing the same proteins from their endogenous loci. 5, 20 and 50% of cells lysates overexpressing the PABPs for 24 hours was loaded for calibration. **B)** Cells over-expressing PABP1-eYFP or PABP2-eYFP were treated with sinefungin (SF) for 60 minutes, with heat shock (41°C for 120 min) or incubated in PBS (120 minutes). Fluorescent microscopy images of a representative cell are shown.
(PDF)

Figure S6 **Distribution of TbPABP1 and TbPABP2 across sucrose gradients.** Cells expressing PABP1-4Ty1 from the endogenous locus were either left untreated **(A)**, treated with cycloheximide or anisomycin **(B)** or puromycin **(C)** for 30 minutes prior to harvesting and during harvesting. Clear lysates were separated on 10–50% sucrose gradients. PABP1-4Ty1 and PABP2, as well as the control proteins BiP and P0 were detected across the fractions of the gradients by quantitative Western blotting. The absorption profile of the sucrose gradient at 254 nm, the western blots and western blot quantifications are shown. In two cases (experiment II of untreated and puromycin treated cells) two separate cell lines were used, each expressing one isoform of PABP as a C-terminally tagged Ty1-fusion protein. Detection of the proteins was done by non-quantitative Western blotting (ECL).
(PDF)

Figure S7 **Changes in PABP1 band-pattern in response to transcriptional and translational inhibitors.** Procyclic trypanosomes expressing PABP1-Ty1 from the endogenous locus were treated with puromycin (PURO; 50 µg/ml), actinomycin D (ACT D; 10 µg/ml), cycloheximide (CHX; 50 µg/ml), heat shock (HS; 41°C) or sinefungin (SF; 2 µg/ml) for the indicated times. Cells were washed in SDM79 without serum and drugs as required and prepared for western blot. 5*10^6^ cell equivalents were loaded and PABP1-4Ty1 and PABP2 were detected by quantitative western blotting. Gels of two biological replicates are shown.
(PDF)

Figure S8 **A+B)** Inducible expression of double tomato fluorescent protein (dTFP) fused to different fragments of the region of PABP2 that lies between RRM3 and RRM4 and contains the predicted NLS **(B)**. A random sequence as well as the NLS of the LA protein [75] served as negative and positive controls, respectively **(A)**.
(PDF)

Figure S9 **Differential localization of translation initiation factors eIF4E1-4 (A) and eIF4G3 (B) to inducible RNA granules.** Both proteins were expressed as eYFP fusions in a cell line also expressing PABP1-mChFP.
(PDF)

Table S1 **Sequences used in the phylogenetic reconstruction of PABP in the Excavates.**
(PDF)

Table S2 **Plasmids used in this work. Some of the plasmids have been previously described** [**63**] **.**
(PDF)
