# Supplementary material for: Differential Localization of the Two T. brucei Poly(A) Binding Proteins to the Nucleus and RNP Granules Suggests Binding to Distinct mRNA Pools
Source: PLoS One. 2013 Jan 30;8(1):e54004. doi: 10.1371/journal.pone.0054004 (PMC3559699; doi:10.1371/journal.pone.0054004)
Supplement: Figure S1 — RNAi knock-down of either TbPABP1 or TbPABP2 is lethal. PABP1 (A) and PABP2 (B) knock-down by tetracycline (TET) inducible RNAi. Both growth (top) and the reduction in PABP1 and PABP2 mRNAs (bottom) were monitored over a time-course of RNAi induction in procyclic cells. RNA from wild type cells (wt) served as control. Loading of the northern blots was controlled by reprobing for ribosomal RNA. (PDF) [file pone.0054004.s001.pdf]

Figure S1

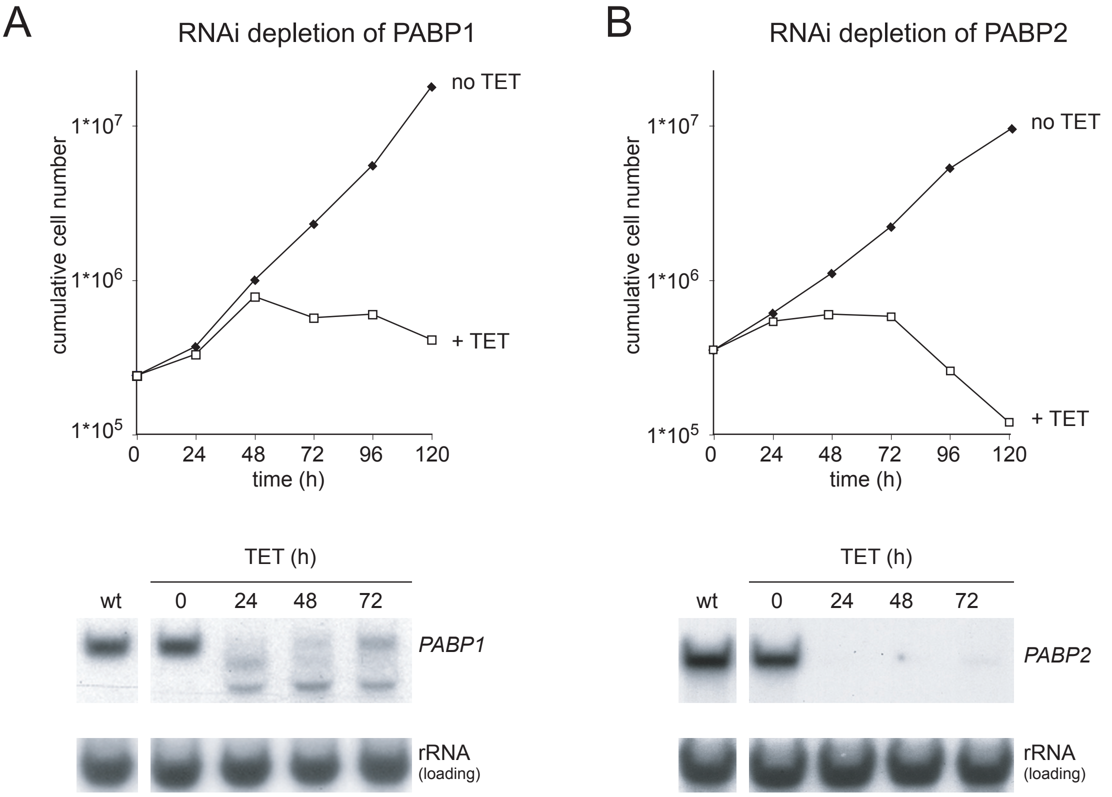

Figure S1: RNAi knock-down of either TbPABP1 or TbPABP2 is lethal
PABP1 **(A)** and PABP2 **(B)** knock-down by tetracycline (TET) inducible RNAi. Both growth (top) and the reduction in PABP1 and PABP2 mRNAs (bottom) were monitored over a time-course of RNAi induction in procyclic cells. RNA from wild type cells (wt) served as control. Loading of the northern blots was controlled by reprobing for ribosomal RNA.
