# Supplementary material for: Differential Localization of the Two T. brucei Poly(A) Binding Proteins to the Nucleus and RNP Granules Suggests Binding to Distinct mRNA Pools
Source: PLoS One. 2013 Jan 30;8(1):e54004. doi: 10.1371/journal.pone.0054004 (PMC3559699; doi:10.1371/journal.pone.0054004)
Supplement: Figure S2 — Verification and analysis of PABP1-eYFP/Δpabp1 procyclic cells. A) To-scale maps of the PABP1 genomic locus: wild type (top), after replacement of the PABP1 gene by a puromycin resistance gene (ΔPABP1, middle) and after integration of PABP1-eYFP (bottom). AgeI restriction enzyme sites and positions of the probes used for the Southern Blot in B) are indicated. B) Verification of the transgenic cell lines by Southern blot. C) Growth curves of wild type cells and transgenic cell lines. Population doubling times (PDT) are indicated. (PDF) [file pone.0054004.s002.pdf]

FIGURE S2

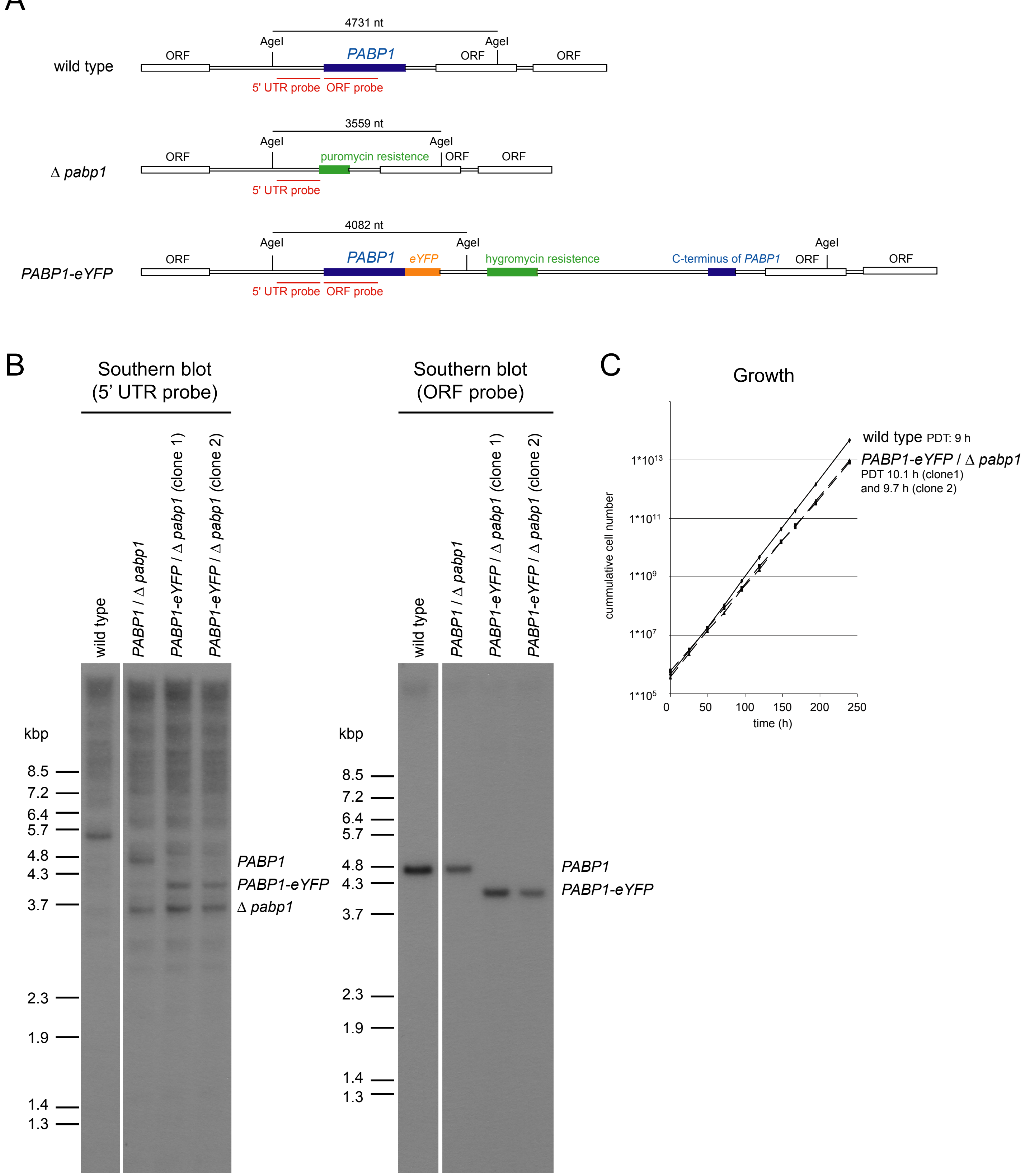

Figure S2: Verification and analysis of PABP1-eYFP / Δpabp1 procyclic cells
A) To-scale maps of the PABP1 genomic locus: wild type (top), after replacement of the PABP1 gene by a puromycin resistance gene (ΔPABP1, middle) and after integration of PABP1-eYFP (bottom). AgeI restriction enzyme sites and positions of the probes used for the Southern Blot in B) are indicated.
B) Verification of the transgenic cell lines by Southern blot.
C) Growth curves of wild type cells and transgenic cell lines. Population doubling times (PDT) are indicated.
