# Supplementary material for: Differential Localization of the Two T. brucei Poly(A) Binding Proteins to the Nucleus and RNP Granules Suggests Binding to Distinct mRNA Pools
Source: PLoS One. 2013 Jan 30;8(1):e54004. doi: 10.1371/journal.pone.0054004 (PMC3559699; doi:10.1371/journal.pone.0054004)
Supplement: Figure S3 — Detection of PABP2 in inducible RNP granules by immunofluorescence. 2*107 cells, untreated or stressed by carbon source starvation (3 hours PBS), 2 h heat shock at 41°C (HS), sinefungin (SF) or 2 hours heat shock and sinefungin (HS+SF) were washed once in SDM79 without serum and heme, resuspended in 5 ml SDM79 without serum and heme and fixed for 15 minutes with 1 volume 8% paraformaldehyde in PBS at RT while rotating. Cells were washed once in PBS and allowed to settle on slides for 15 minutes. Slides were washed in 25 mM NH4Cl for 10 minutes. Cells were permeabilized and blocked for one hour in blocking solution (taken from fluorescent antibody enhancer set for DIG detection, Roche) containing 0.5% saponin. Slides were washed, blocked for an additional 30 minutes without saponin, incubated with the first antibody (anti-LmPABP2 1:500 or anti-GFP, Invitrogen A11122, 1∶100) for 60 minutes, washed 4 times in PBS, incubated with the secondary antibody (anti-mouse Alexa 568) and mounted in FluorSave (Calbiochem), containing Höchst33342 DNA stain at 5 µg/ml. Wild type cells stained with anti-GFP or cells stained with no antibody served as controls and showed significantly less signal (not shown). (PDF) [file pone.0054004.s003.pdf]

Figure S3

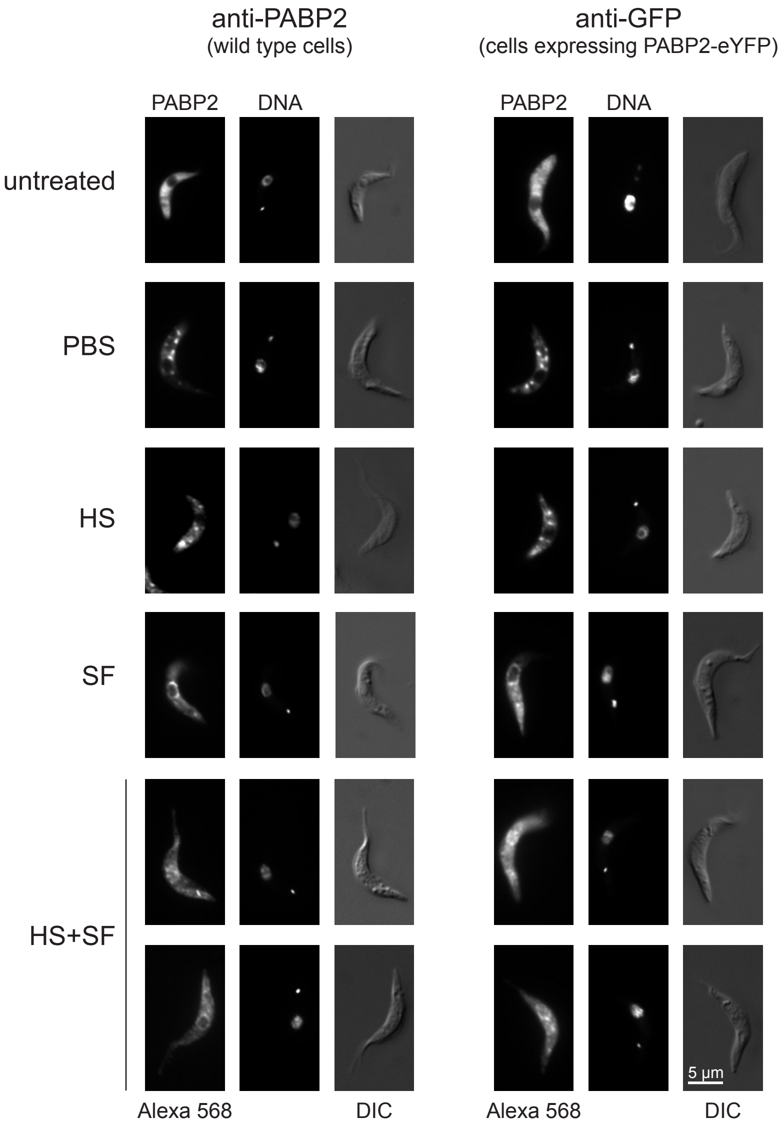

Figure S3: Detection of PABP2 in inducible RNP granules by immunofluorescence
2*10^7 cells, untreated or stressed by carbon source starvation (3 hours PBS), 2 h heat shock at 41ºC (HS), sinefungin (SF) or 2 hours heat shock and sinefungin (HS+SF) were washed once in SDM79 without serum and heme, resuspended in 5 ml SDM79 without serum and heme and fixed for 15 minutes with 1 volume 8% paraformaldehyde in PBS at RT while rotating. Cells were washed once in PBS and allowed to settle on slides for 15 minutes. Slides were washed in 25 mM NH_4Cl for 10 minutes. Cells were permeabilized and blocked for one hour in blocking solution (taken from fluorescent antibody enhancer set for DIG detection, Roche) containing 0.5% saponin. Slides were washed, blocked for an additional 30 minutes without saponin, incubated with the first antibody (anti-LmPABP2 1:500 or anti-GFP, Invitrogen A11122, 1:100) for 60 minutes, washed 4 times in PBS, incubated with the secondary antibody (anti-mouse Alexa 568) and mounted in FluorSave (Calbiochem), containing Höchst33342 DNA stain at 5 µg/ml. Wild type cells stained with anti-GFP or cells stained with no antibody served as controls and showed significantly less signal (not shown).
