# Supplementary material for: Differential Localization of the Two T. brucei Poly(A) Binding Proteins to the Nucleus and RNP Granules Suggests Binding to Distinct mRNA Pools
Source: PLoS One. 2013 Jan 30;8(1):e54004. doi: 10.1371/journal.pone.0054004 (PMC3559699; doi:10.1371/journal.pone.0054004)
Supplement: Figure S4 — Localization of PABP1 and PABP2 in response to heat shock (2 hours at 41°C). A) Cells expressing PABP1-eYFP and PABP2-mChFP. B) Cells expressing PAPB2-eYFP. C) Cells expressing PABP1-eYFP. All fusion proteins were expressed from their endogenous loci. (PDF) [file pone.0054004.s004.pdf]

Figure S4

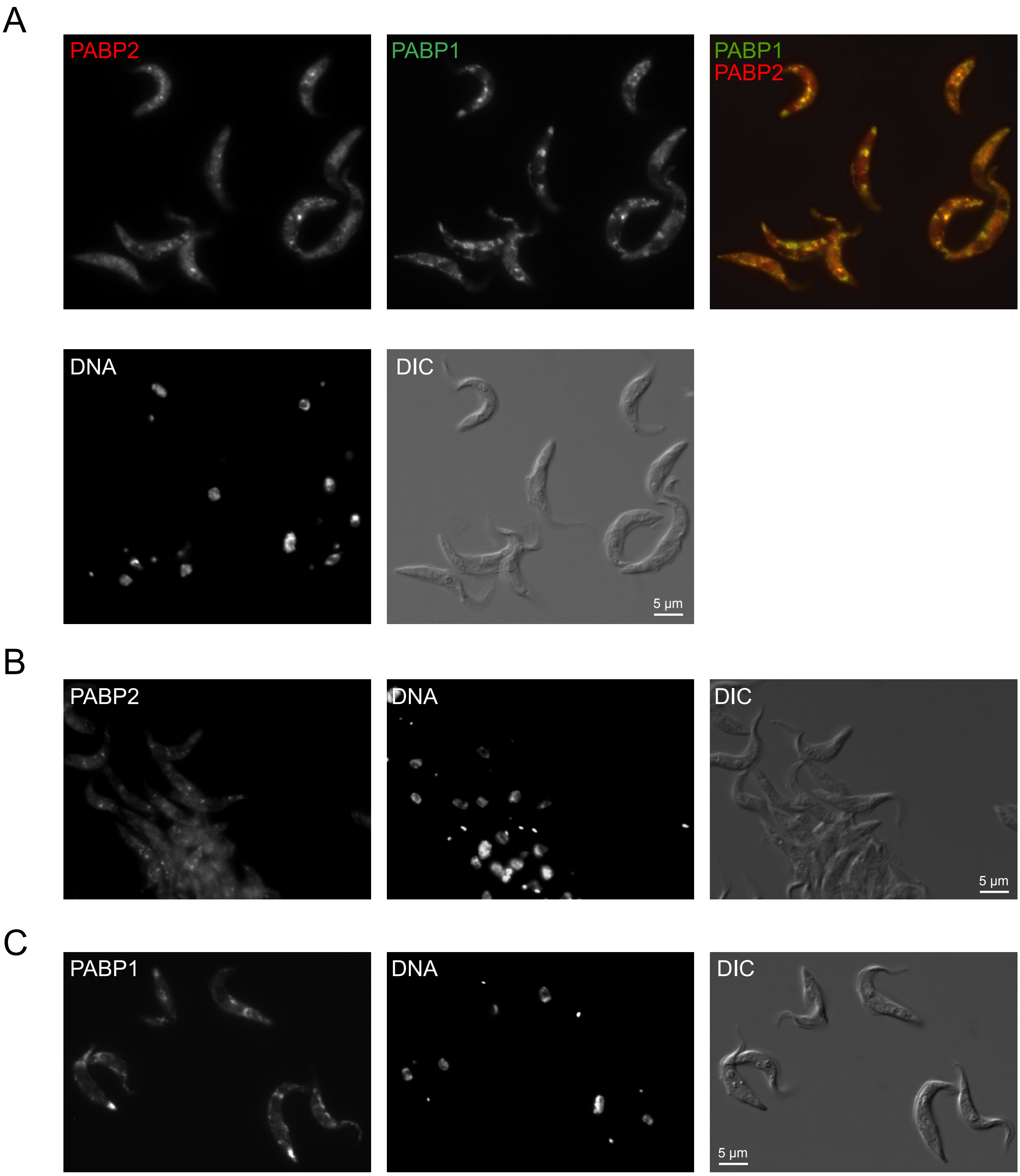

Figure S4: Localization of PABP1 and PABP2 in response to heat shock (2 hours at 41ºC)
A) Cells expressing PABP1-eYFP and PABP2-mChFP
B) Cells expressing PAPB2-eYFP
C) Cells expressing PABP1-eYFP
All fusion proteins were expressed from their endogenous loci.
