# Supplementary material for: Differential Localization of the Two T. brucei Poly(A) Binding Proteins to the Nucleus and RNP Granules Suggests Binding to Distinct mRNA Pools
Source: PLoS One. 2013 Jan 30;8(1):e54004. doi: 10.1371/journal.pone.0054004 (PMC3559699; doi:10.1371/journal.pone.0054004)
Supplement: Figure S5 — Inducible over-expression of PABP1-eYFP and PABP2-eYFP. A) Western blots: 5*106 cell equivalents of cells induced to over-express PABP1-eYFP or PABP2-eYFP for 24 or 48 hours (TET) or cells expressing the same proteins from their endogenous loci. 5, 20 and 50% of cells lysates overexpressing the PABPs for 24 hours was loaded for calibration. B) Cells over-expressing PABP1-eYFP or PABP2-eYFP were treated with sinefungin (SF) for 60 minutes, with heat shock (41°C for 120 min) or incubated in PBS (120 minutes). Fluorescent microscopy images of a representative cell are shown. (PDF) [file pone.0054004.s005.pdf]

Figure S5

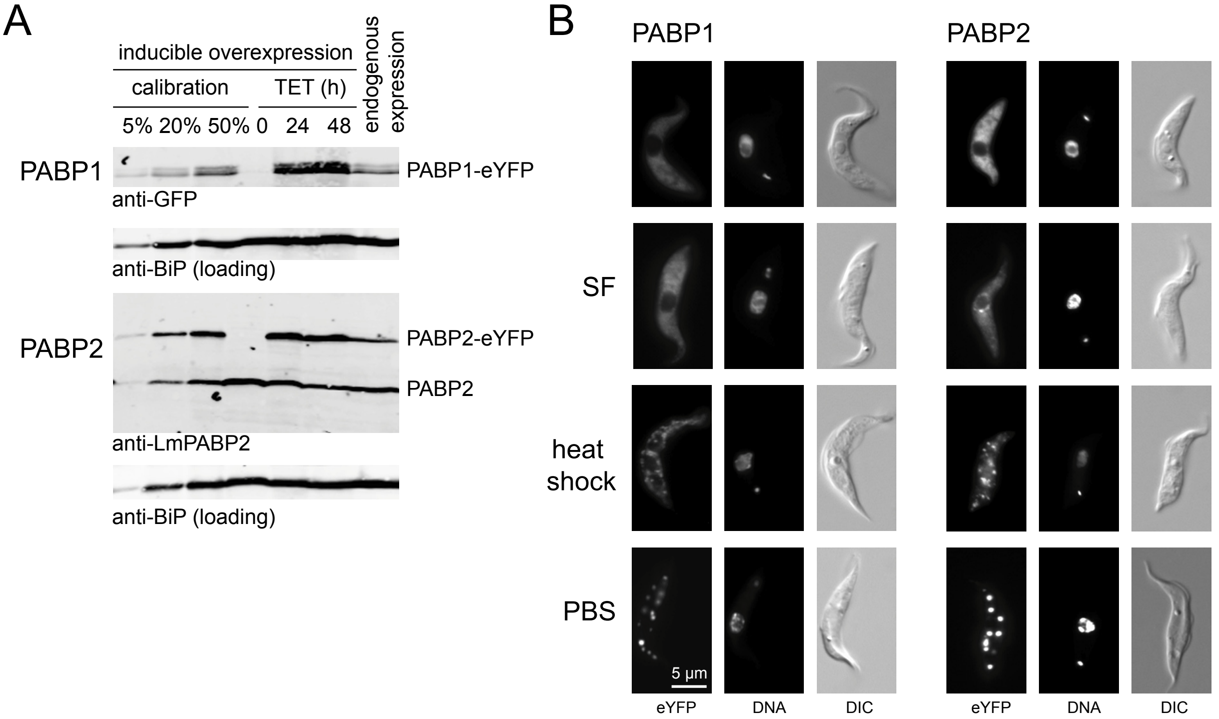

Figure S5: Inducible over-expression of PABP1-eYFP and PABP2-eYFP

A) Western blots: $5*10^6$ cell equivalents of cells induced to over-express PABP1-eYFP or PABP2-eYFP for 24 or 48 hours (TET) or cells expressing the same proteins from their endogenous loci. 5, 20 and 50% of cells lysates overexpressing the PABPs for 24 hours was loaded for calibration.

B) Cells over-expressing PABP1-eYFP or PABP2-eYFP were treated with sinefungin (SF) for 60 minutes, with heat shock (41ºC for 120 min) or incubated in PBS (120 minutes). Fluorescent microscopy images of a representative cell are shown.
