# Supplementary material for: Differential Localization of the Two T. brucei Poly(A) Binding Proteins to the Nucleus and RNP Granules Suggests Binding to Distinct mRNA Pools
Source: PLoS One. 2013 Jan 30;8(1):e54004. doi: 10.1371/journal.pone.0054004 (PMC3559699; doi:10.1371/journal.pone.0054004)
Supplement: Figure S6 — Distribution of TbPABP1 and TbPABP2 across sucrose gradients. Cells expressing PABP1-4Ty1 from the endogenous locus were either left untreated (A), treated with cycloheximide or anisomycin (B) or puromycin (C) for 30 minutes prior to harvesting and during harvesting. Clear lysates were separated on 10–50% sucrose gradients. PABP1-4Ty1 and PABP2, as well as the control proteins BiP and P0 were detected across the fractions of the gradients by quantitative Western blotting. The absorption profile of the sucrose gradient at 254 nm, the western blots and western blot quantifications are shown. In two cases (experiment II of untreated and puromycin treated cells) two separate cell lines were used, each expressing one isoform of PABP as a C-terminally tagged Ty1-fusion protein. Detection of the proteins was done by non-quantitative Western blotting (ECL). (PDF) [file pone.0054004.s006.pdf]

Figure S6

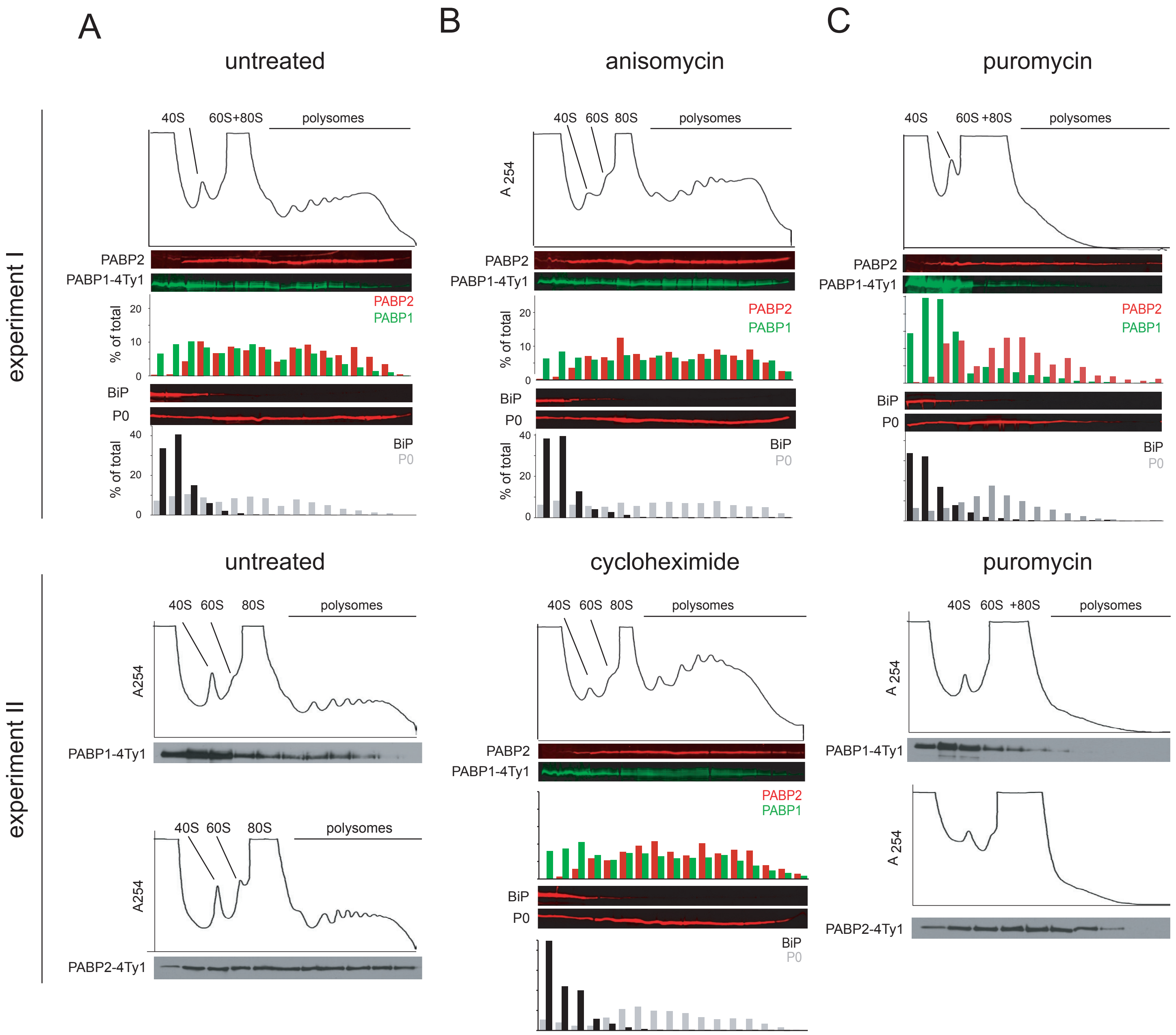

Figure S6: Distribution of TbPABP1 and TbPABP2 across sucrose gradients
Cells expressing PABP1-4Ty1 from the endogenous locus were either left untreated **(A)**, treated with cycloheximide or anisomycin **(B)** or puromycin **(C)** for 30 minutes prior to harvesting and during harvesting. Clear lysates were separated on 10-50% sucrose gradients. PABP1-4Ty1 and PABP2, as well as the control proteins BiP and P0 were detected across the fractions of the gradients by quantitative Western blotting. The absorption profile of the sucrose gradient at 254 nm, the western blots and western blot quantifications are shown. In two cases (experiment **II** of untreated and puromycin treated cells) two separate cell lines were used, each expressing one isoform of PABP as a C-terminally tagged Ty1-fusion protein. Detection of the proteins was done by non-quantitative Western blotting (ECL).
