# Supplementary material for: Differential Localization of the Two T. brucei Poly(A) Binding Proteins to the Nucleus and RNP Granules Suggests Binding to Distinct mRNA Pools
Source: PLoS One. 2013 Jan 30;8(1):e54004. doi: 10.1371/journal.pone.0054004 (PMC3559699; doi:10.1371/journal.pone.0054004)
Supplement: Figure S7 — Changes in PABP1 band-pattern in response to transcriptional and translational inhibitors. Procyclic trypanosomes expressing PABP1-Ty1 from the endogenous locus were treated with puromycin (PURO; 50 µg/ml), actinomycin D (ACT D; 10 µg/ml), cycloheximide (CHX; 50 µg/ml), heat shock (HS; 41°C) or sinefungin (SF; 2 µg/ml) for the indicated times. Cells were washed in SDM79 without serum and drugs as required and prepared for western blot. 5*106 cell equivalents were loaded and PABP1-4Ty1 and PABP2 were detected by quantitative western blotting. Gels of two biological replicates are shown. (PDF) [file pone.0054004.s007.pdf]

Figure S7

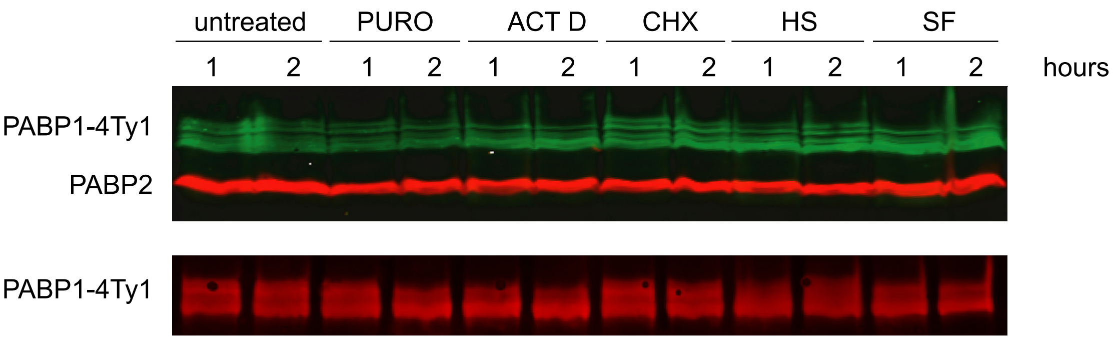

Figure S7: Changes in PABP1 band-pattern in response to transcriptional and translational inhibitors
Procyclic trypanosomes expressing PABP1-Ty1 from the endogenous locus were treated with puromycin (PURO; 50 µg/ml), actinomycin D (ACT D; 10 µg/ml), cycloheximide (CHX; 50 µg/ml), heat shock (HS; 41ºC) or sinefungin (SF; 2 µg/ml) for the indicated times. Cells were washed in SDM79 without serum and drugs as required and prepared for western blot. $5*10^6$ cell equivalents were loaded and PABP1-4Ty1 and PABP2 were detected by quantitative western blotting. Gels of two biological replicates are shown.
