# Supplementary material for: Differential Localization of the Two T. brucei Poly(A) Binding Proteins to the Nucleus and RNP Granules Suggests Binding to Distinct mRNA Pools
Source: PLoS One. 2013 Jan 30;8(1):e54004. doi: 10.1371/journal.pone.0054004 (PMC3559699; doi:10.1371/journal.pone.0054004)
Supplement: Figure S8 — A+B) Inducible expression of double tomato fluorescent protein (dTFP) fused to different fragments of the region of PABP2 that lies between RRM3 and RRM4 and contains the predicted NLS (B). A random sequence as well as the NLS of the LA protein [75] served as negative and positive controls, respectively (A). (PDF) [file pone.0054004.s008.pdf]

Figure S8

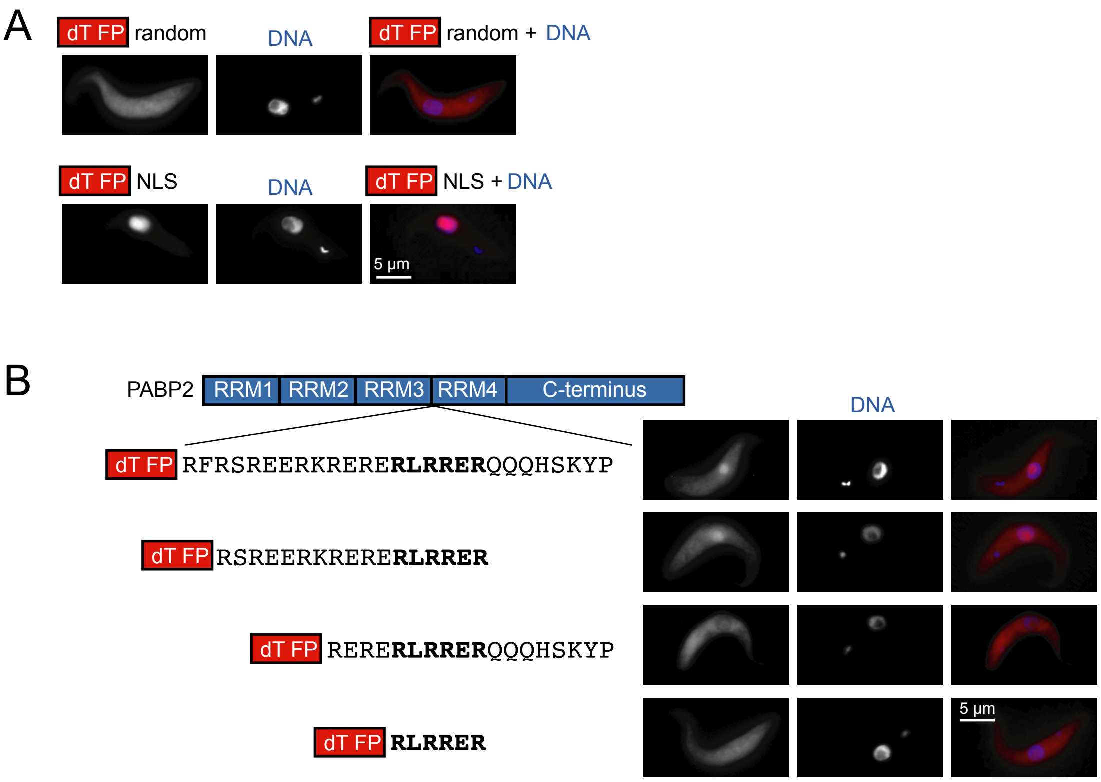

Figure S8
A+B) Inducible expression of double tomato fluorescent protein (dTFP) fused to different fragments of the region of PABP2 that lies between RRM3 and RRM4 and contains the predicted NLS **(B)**. A random sequence as well as the NLS of the LA protein (Marchetti et al., 2000) served as negative and positive controls, respectively **(A)**.

Marchetti MA, Tschudi C, Kwon H, Wolin SL, Ullu E (2000) Import of proteins into the trypanosome nucleus and their distribution at karyokinesis. J Cell Sci 113 (Pt 5): 899-906.
