# Supplementary material for: Differential Localization of the Two T. brucei Poly(A) Binding Proteins to the Nucleus and RNP Granules Suggests Binding to Distinct mRNA Pools
Source: PLoS One. 2013 Jan 30;8(1):e54004. doi: 10.1371/journal.pone.0054004 (PMC3559699; doi:10.1371/journal.pone.0054004)
Supplement: Figure S9 — Differential localization of translation initiation factors eIF4E1-4 (A) and eIF4G3 (B) to inducible RNA granules. Both proteins were expressed as eYFP fusions in a cell line also expressing PABP1-mChFP. (PDF) [file pone.0054004.s009.pdf]

Figure S9 A

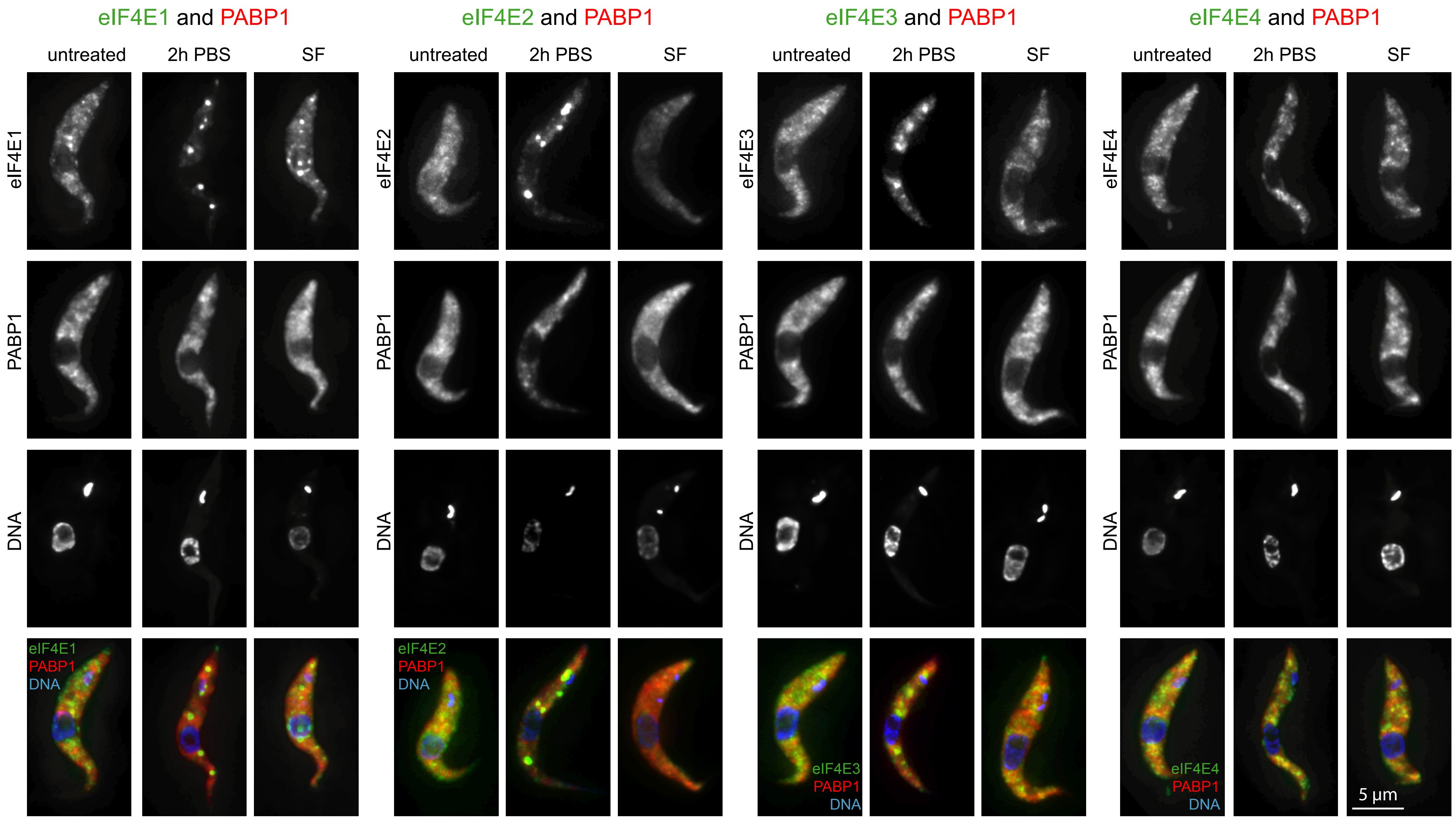

eIF4E1 and PABP1
eIF4E2 and PABP1
eIF4E3 and PABP1
eIF4E4 and PABP1
untreated
2h PBS
SF
eIF4E1
eIF4E2
eIF4E3
eIF4E4
PABP1
DNA
eIF4E1
PABP1
DNA
eIF4E2
PABP1
DNA
eIF4E3
PABP1
DNA
eIF4E4
PABP1
DNA
5 µm

Figure S9B

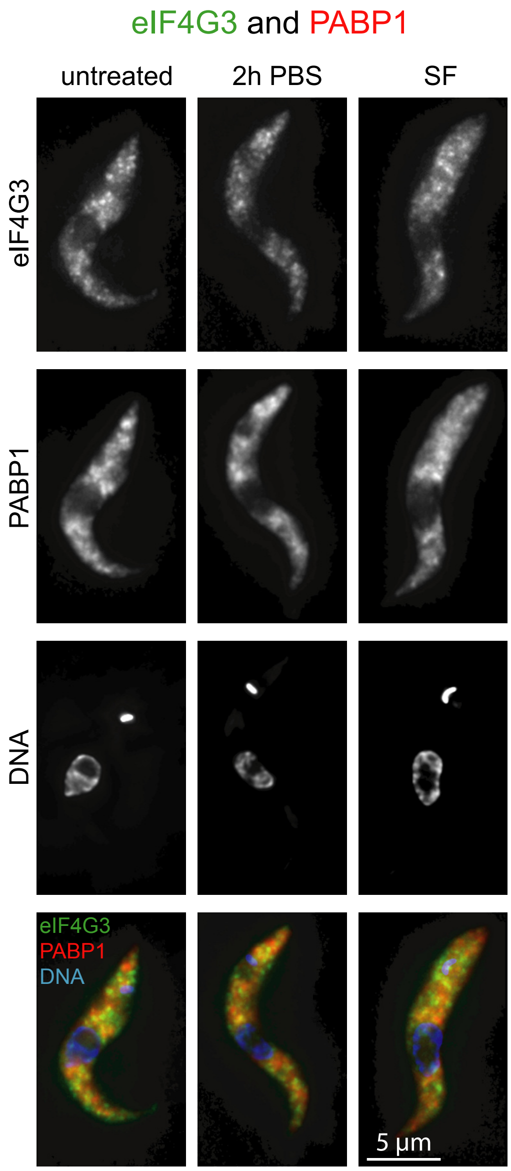

Figure S9: Differential localization of translation initiation factors eIF4E1-4 (A) and eIF4G3 (B) to inducible RNA granules.
Both proteins were expressed as eYFP fusions in a cell line also expressing PABP1-mChFP.
