# Supplementary material for: Differential Localization of the Two T. brucei Poly(A) Binding Proteins to the Nucleus and RNP Granules Suggests Binding to Distinct mRNA Pools
Source: PLoS One. 2013 Jan 30;8(1):e54004. doi: 10.1371/journal.pone.0054004 (PMC3559699; doi:10.1371/journal.pone.0054004)
Supplement: Table S1 — Sequences used in the phylogenetic reconstruction of PABP in the Excavates. (PDF) [file pone.0054004.s010.pdf]

Table S1: Sequences used in the phylogenetic reconstruction of PABP in the Excavates.

>Tthe1
MTTATQEEITPTVPVTKPLQVASLYVGDLDPAINEPQLVELFKPYGTILNVRVCRDIITQRSLGYGYVN
FDSPESAAKAMESLNFKRIGEKCVRIMWQQRDPALRYSGNGNIFVKNLKNEVDSRALSLIFKKFGDILS
CKVMEDEGGKSRGYGFVHFKEDDAAKDAITNMNGDKENADEAKSGLYVANFIRRNARLAALVANFTNVY
IKQVLPTVDKDIIEKFFSKFGGITSSATCKDKNGRVFAFCNFEKHDDAVKAIEAFHDQVIEGVTAPGEK
LYVQRAQPRSERLIALRQKYMQCQSLGNNLYVRNFDPDFTEKNLHELFQEYGEIRSCRVMTDANGNSRG
FGFVSFENADQANAALREMNGRMLNGKPLIVNIAQRRDQRYTMLRLQFQQRLQMMMRRMHPAPFVAQGP
GPQRRNARNAQRGGSGGGAGRHHQAPQQAPAPHPQPDMFSTPSMAFTAQRTPQPSPGVAPDTPPLPPIT
AEDLRTMTVDEQRAALGDRLYIKVFEIAPDHAPKITGMFLEMDLKEAFTLLYNQKLLHEKVTEALCVLK
AHGTTA

>Tthe3
MDAPLAQRTSSLYVGDLPVEMNNPEEVLGRLFGSVGPVASIRVCRDMNTQRSLGYAYVNFQNPADAEKA
IESFNYTDIIPGHSIRIMPSMRDPILRRAGINNLYVKIADRSLTARDLRDIFSRYGKILSTKVTCDTNG
KSLGYGFVQFETAEGAKTALEKSVESLGEAIIAVAPFVRKAERLAQEEKKFVNVYVKNIKPGTTEDEIR
GEFNKFGELKSLFLSGHPQHQTMFCILSFLTHDAAVAAIEAMHNSVSSPILLPDSKLFVCRALKKRDRQ
EIAQSSQTLYQSQGRNLYIKHLDESVTREKLEELFSPFGKIVSCVLMKDASGVSREFGFVCFEAKEIAD
TALREMNGKTVFGKPLYVSYAEQKDMRSRILHEKIQRMIRQQHQRTTMPMTILSKPWTRNYPANSVFRP
PFMQPFHARRQPPHPPPPMFMTSSMQTPPPPPPPVPSQLMNHGTNRTYYTQPHFPVPPLPPPPPPPPI
HSAQQVWQGANGEALRESHLSNLSPEEKRNILGERLFSKVMEIQPNQAAKITGMLLEMETAEILEILNT
SSLLTAKVEEAIVVLRQHTNRV

>Tthe2
MSTFPAASPSIWVGGLDPNLNEQKLYDHFVRVGPVASVRVCVDSATQKSLGYGYVNFQNPADAEKALDQ
AGVKLGSKHIRIAKIQRDPSKRRSGVTNIVVKKLPPAVDTYALKEMFSKFGRLTAIGLATNENGESRGY
ARISFEKEESAVEAVQEVNGMTIDDCVITVERYQPHHREEQLKQYTNLYVKNLDPSVDDEKLKETFSAF
GEVTSAKVRNLGPSQTVGFAYVAYATHEAAAKAVEELDEKESPLAKEGLKLSVCRFRSREERQRERERL
RRERQQQHSKYPNLYVKNFDDTVTSSRLKEVFERFGETVSVSVMMDKATKLSRCFGFVSFKEQSSAARA
IQELHGSTVLGPRPLFVTYALRKDARRQTLEDIRNKQPRMRQPPMGGLMGGMMGPQLGFMGPQTMFNGV
PFVNPRMPMMPATMGMGAPIRPLGPTPMNQVRARPAPQRVPMQQMMAAPQPQPPAPQGQNLSTVLANLT
PEQQKNVLGERLYNYIVRVNPSVAAKVTGMLLEMDNSEILNLLDNPALLDTKVQEALDVLNRHMNV

>Tcar1
MTTSTDEEVIQAVPIANTLRVASLYVGDLDPVIDEPQLVEIFKSYGTILNVRVCRDIITQRSLKYGYVN
FDSHESAAKAMEELNFKRFGEKCIRIMWQQRDPVLRYSANGNIFVKNLKNEVDSGQLHLLFKKFGDILS
CKVMEDEEGKSRGYGFVHFKEDDSAKKAIENMNGDKEIADKEKEALYVANFIRRNLRLAALVANFTNVY
IKQVLPTVDKDIIEKFFSKFGGITSSAALKDKNGRVFAFCNFQNHDDAVKAIEAFHDQNVDGITPPGEK
LYVQRAQPRSERLIALRQKYMQCQSLGNNLYVRNFDLEFTDDNLYELFKEYGVIKNCRVMTDANGNSRG
FGFVSFENAEQANAALREMNGRMLNGKPLIVNVAQRRDQRYTLLRVQFQQRLQMMMRRMHSVPFALHGP
PQQPQHSRGRMGRGAPRGTRNHAPLHTPPPTQSQVQAQAQAQTHVQEMFTTPSMGFAAPRTPQASPGVA
PETPPLPPITADDLSHMSTDEQRAALGDRLYIKVFEMAPDHAPKITGMFLEMDLKEAFALLSNQKLLHE
KVTEALCVLKAHGSAA

>Tcar2
MSSFPKESPSIWVGGLDPNLNEQKLYDHFVHVGPIVSVRVCVDSVTQKSLGYGYVNFQNLSDAEKALDQ
AGMKLGSKHIRVAKIQRDPSKRRSGVTNIVVKKLPHTLDTEYLKQMFTPFGRLTAIGLATDEKGESRGY
ARISFEKEESAIQAAQEMNGTTIEDSVITVERYQPHHREEQLKQFTNLYVKNLDSSVDDEKLKEVFSKF
GEVTSAKVREIGQNPGAGFAYVAYATHEAAASAVEELDEKESPLAKEGMKLSVCRFRSREERHREHERL
RRERQQQHSKYPNLYVKNFDDTVTDERLKELFERFGETVSVRVMIDMATKTSRCFGFVSFKEQSSAARA
VQELHGSTYLGPRPLFVTYALRKDARRQTLEDMRNKQPRMRQPPIGGMMGGMMGAQIGFMNPQSMFNGV
PLMNPRMPMMPAAMGMGAPIRPLGPTPMNQVRARPAPQRPLMQPLMQPQQPQAPAQGQNLSTVLASLSP
EQQKNVLGERLYNYIVRVNPSVAAKVTGMLLEMDNGEILNLLDNPSLLNTKIQEALDVLNRHMSV-
RTTNGLAAGWRSSMECNPLQDRNHIGEDVFRK

>Tbor1
MSVEEITPTTQVIEQKPSITSIYVGDLSPEVDEPDLLELFKTCGIVLGVRILRDILTRRSLGYGYVNFQ
NAAEAEKALETLNFKTVKNCNIRLMYQQRDPTTRYNGSGNIFVKNLDGKVDSKSLHEIFNKFGSILSCK
VMSNADGSSRGYGFVHFKDDSQASEAICKMNGNNENAAENAALYVAQFIRRNNRIAALMANFTNVYIKQ
VLPNVDKDIIEKFFSKFGGITSACAKQDSKGRTFAFCNFSNHEEAVKAIEEVHDKPIDGLTSPDDSIYV
QRAQPRNERLVELRAKSMQRQSLGNNLYVRNFGPNFTEKQLRELFGEYGDIKSCRIMTDDKGTSRGFGF

VSFVTAEQANIALRELNGRLFDGKPLVVNIAQRRDQRFVMLQAQFQQRIQALMQQLPVMPFIAGGSQQG
GRRQPNRKTTPMPQQLTTAPKRQLVRRGVEQYPSASRTPMSSPAMVPMTPTLAPITEEDLLKMPINEAK
SALGERLFIKISAINSELAPKITGMFLEMDIREGFALLQNAEDLKDKVNEAMYVIHNHQNSK

>Tbor3
MDVNGPARISGSLYVGDLPVDLHNPEDCLFATFSKIGMVISIKVCRDMNTQRSLGYAYVNFQNPGDAER
ALRELNYSKIRPDHPDEMRVMWTNRDPNNRKHGSGNVFLKNLPTNMTNKELFEFCSKFGHVVSCKHAKK
NGEYHGYGFVDFGTEEVAKKAIEELDGKVYNGHKIEGKPFKRRLERQRDADKTFVSIYCKNIKSDATRE
EVIAFFSKTGPVVDVYVGQPTSYPTKYAIIEMATHEDAVRVIETVSDPDFIISAPETKLFVSRLLKKAE
RIQTTRSVYQNEGRNLYVKNLPDNISEDVLVEMFAPFGPIQSTHLSRDSEGRFRGFAFVCFENKESADK
ACRNLMNELKFGRPLYVAVAQHKDSRHMMLDEQFKNRIMAHSMEMRHNMFPPQVRYPMPMNMNGMPHMA
QMGPMNPMMGGFNQIRRNPGQGRPRGGGRIAPRQHNFQQPHTTHSDISALLSMNPADRKNILGERLYSK
IHPSNPALAAKITGMLLEMETEEILSVLDNDHMLQSKVNEAMRVLEQHNQS

>Tbor2
MAQIPSVSVYVGNLDAVIDERALFEHFKEIGPIVSVRVCLDAQTQRSLRYGYVNFQNTNDAESAIEKLN
LTKLGVEYIHVNHVERDPTKRRAGKSNLVIKNLPENIEVPTLKELFSKFGLITSIKLPQIKETGKTVGF
GYVMFDTIESAVNATEELNGLELEGKTLKVIPYEHDFQEKKRRQFTNLYIRGLRENVSEEQLSAELEKY
GRPTSVAVMPDKYAESSAKGFAFAAFETHEGAVAAIEALNDKVTPSLCDPDCPLQVARFKSRSERYKDA
ESRKLARRLEFSKYPNLYVKNFGDDVDSEKLKSIFDRFGETVSVRVMIDSKTGLSRLFGFVSFKEGAAA
DRCIAELNGSTIFSNRPLYVARAVKKDARQRQLEEEVRNNSLMNNMHNRFPAHHHNQMMAPNMMYNRPN
PNNQMGMMMNQQQRTHNPRPQRTAYPQHQHQHHQHQQHHHQHQQHHHHHHMQQMQHQQMQQQQHMQRMQ
QMHQPQTKSLASILAAIPPEQQHNMLGERLYSHICIRHPNEAAKVTGMLLEMEMSEILNLLEDVDLLEK
KIEEALGVLINHQSIG

>Tgra1
MTATQEEVAPEVSIKKPLQVASLYVGDLDPAINEPQLVELFKPYGTILNVRVCRDIITQRSLGYGYVNF
DSNESASKAMEALNFKRIGEKCIRIMWQQRDPALRYSGDGNIFVKNLNNEVDSRELSIIFKKFGDILSC
KVMEDEGGKSRGYGFVHFKDDDAAKNAIEKMNGAKDYADAEKAGLYVANFIRRNARLAALVANFTNVYI
KQVLPTVDKEIIEKFFSKFGGITSAATCKDKNGRVFAFCNFEKHDDAVKAIEAFHDQVVEGVTPPGEKL
YVQRAQPRSERLIALRQRYMQCQSLGNNLYIRNFDPDFTEENLRELFKEYGEIRSCRVMADANGNSRGF
GFVSFENADQANAALREMNGRMLNGKPLIVNIAQRRDQRYTMLRLQFQQRLQMMMRRMHPMPFASQGPT
PQRRNMRGAQRGGGNRHQAPLHSAPPPQHMQQQQQMQQSDMFGTPSMAFAAPRTPQPSPGAAPDTPPLP
PITAEDMRTMTVDEQRAALGDRLYIKVFEIAPDHAPKITGMFLEMDLKDAFTLLYNQKLLQEKVTEALC
VLKAHESTA

>Tgra2
MSAFPAASPSIWVGGLDPNLNEQKLYDHFVRIGPVASVRVCVDSVTQKSLGYGYVNFQNPADAEKALDQ
AGVKLGSKHIRIAKIQRDPSKRRSGVTNIVVKKLPPTVDTYALKEMFSRFGRLTAIGLATNEHGDSRGY
ARISFEKEESAVEAVQEVNGMVIDDCTITVERYQPHHREEQLKQFTNLYVKNLDPSVDDEKLKEVFSAY
GEVTSAKVRDLGANQTVGFAYVAYATHEAAAKAVEELDEKESPLASEGQKLSVCRFRSREERERERERL
RRERQQQHSKYPNLYVKNFDDTVTSSRLKELFERYGETVSVWVMMDKATKMSRCFGFVSFKEQSAASRA
IQELHGSTILGPRPLFVTYALRKDARRQTLEDMRNKQPRMRQPPMGGLMGGMMGPQLGFMGPQTMFNGV
PFANPRMPMMPATMGMGGPMRPLGPTPMNQVRARPAPPRPAMQPMVAPPPQPPAPAPQGQNLSTMLANL
TPEQQKNVLGERLYNYIVRVNPSVAAKVTGMLLEMDNSEILNLLDTPGLLDTKVQEALDVLNRHMSV

>Tgra3
MDGPLSQRTASLYVGDLPVDMSNPEEALGRVFGSVGPVASIRVCRDMNTQRSLGYAYVNFQHPADAEKA
LETLNYTDIIPGRPIRIMPSMRDPTMRRTGANNVYVKISDKSWTAKDLRDVFSSCGKVLSAKVTCDSDG
KSLGYGFVLFESADAAKAALNMGVDSLGDGVAAVAPFIRKMERLAEEAKTFVNVYTKNIKKGEAEENVR
KEFENFGEVTSMFLSGHPQHETMFSIVSFATHDAAVAAINSLNGSADSPIAIPDGKLFVCRALKKKDRQ
QAEASGQALYQSQGRNLYVKHLDDSVTREKLEELFSPFGKITSCALMRDASGVSREFGFVCFETKEDAA
AALREMNSKTVYSKPLYVSYAEQKDMRRRILHEKMQRVLRQQQQQHRPPVPMTMFNQPWSRNYPVNPT
FRPPFMQPPNARRPPPPMFMPQTMPPPPPLMMNQGTNRPHYTPPHSSLPPPPPPPPPPMQQPVRHLWQK
ANGEELQPDQIAKLSPEEKRNILGERLFGKVVEIRPNQAAKITGMLLEMETAEILEILDVPSLLMAKVE
EAVVVLRQHSNGV

>Pdav1
MTTVHQQDEAQQAAVASKPFQVASIYVGDLDATITEPQLVELFKPFGTILNVRVCRDIITQRSLGYGYV
NFDSHGDAEKAIETLNFKRVGDKCMRLMWQQRDPALRYSGNGNIFVKNLEGEVDSKGLHDIFQKFGNIL
SCKVMEDEEGKSRGYGFVHFKDETAAKSAIEKMNGAKDHANAEKQALYVANFIRRNARLAALVANFTNV
YIKQVLPTVDKAVIEKFFAKFGGITSAAACKDKNGRVFAFCNFAKHDDAVKAIEAMHDQSIDGISAPGE

KLYVQRAQPRSERLIGLRQKYMQSQSLGNNLYVRNFDPDFTDASLRELFKEYGDIKSCRVMTDANGASR
GFGFVSFSNADEANAALREMNGRMLNGKPLIVNIAQRRDQRYNMLRMQFQQRLQMMMRQIHQQQQQQMP
FVAGPSQQQQQPRGGMRGRSGRHQQGRGHGQPMPMPSPQQQPAFGTPSAAGLASPTPKQSPGTAPETPP
LPPISPEELFAMSPDEQRAALGDRLYIKVYEMSPEYAPKITGMFLEMEMKEAYELLNNHKLMTDRVTEA
LCVLKAHDRA

>Pdav3
MAVGPQRTSLYVGDIPIDLPHPEDAINSLFNSVAPVVSLRVCRDMNTQKSLGYAYVNFQNNTDAEKALD
ALNFTEIVPGREIRVMFSIRDPIIRKSGANNLCVKKLDPSVTAKTLHNTFMTFGRVLSSKIASDENGKS
KGYGFVQFETPEAAKAALDMDGKKIGASEVVVVPYMRRSEREAELEKTFTNVFAKNISAKVSDDDVKAA
FEKFGEIETIFVSTHAQHETKFALVTFAKHESAVESIEKLNGNTEAPLADGEAALYVCRAMKKSERSRM
KRKAPSLYQSQGRNLYVKHLPDSITKEELENLFTPFGKITSCALMKDSTSGVFRGFAFVCFDEKESAVA
AMREMNGKSLPDSKNPLYVSQAEQKDMRLRLLQQRRAAFKNQQRMAPPMGMFGQQWGRQYPMMAPMYGA
MPVPPPFLGAPMPPMGRRAMPPMPPPQRPLNMHNMHNMHNMHNMPNRYTQPRPQPQPMNHQPASGSGLD
AAQLAAMGNEERNNLLGETLYNRIVTMNPQQAAKITGMLLEMGTTEILEVLEDENALRAKVSEAISVLQ
HHTTN

>Pdav2
MAFSGPNPSIWVGGLDPELTETNLYEYFIRIGPVASVRVCVDATTQKSLGYGYVNFQDPADAEKALDQA
GSKLGSSY LRVAKVQRDPAKRRAGADNIVVKKLPASIDTFALKEMFSKFGRLTAIGLACDEKGESRGY
ARVSFEREESAADAVKEMNGMEVDGQAIVVERFQPQHRDELLKKYTNLYVKNLNPAITSDELREFFNKY
GETTSAKVHDVVSSKSDVGFGYVAFDKHEDAAKAVEELNGKECEISKEGLTLDVSRFRSREERQRDRER
QRRERAQQFSKYPNLYVKGFDDSVTSDRLKELFERFGETVSVTVMFDRDSGLSRCFGFVSMRDQNAASK
AIQELNGSTFLSPRPLFVTYALRKDARRANLEERSKQHMRMRNQGAGPMNMPPMGFMGQQMFPGMNMPF
MNPRMPMMPMGGAMGPGPMRPMGPMGGPNPMNQMRAGRPMAQRPPVQPMIPPQHQPPTQSQNLSSVLAN
LNPDQQKNVLGERLYTHIVRSHPSLAAKITGMLLEMDNGEILNMLDNPALLDSKIAEAQDVLNRHMNVD
SL

>Pser1
MATAQVETPAQPEQPIQIASVYVGDLDQNITEPQLVDLFKSFGTILNVRVCRDIITQRSLGYGYVNFNS
HEDAKKAIETLNFKRVGDKCIRLMWQQRDPALRYSGSGNIFVKNLGEGVDSKALNDIFQKFGAILSCKV
MVDEQGKSRSYGFVHFKDENAAKTAIEQMNGSTEHANAEKHALYVANFIRRNARLAALVANFTNVYIKQ
VLPTVDKEIIESFFSKFGGITSATTCKDKSGRVFAFCNFEKHDEAVKAIEALHDNHVEGITAPGEKLYV
QRAQPRSERLIALRQKYMQFNSLGNNLYVRNFDPNFTSDNLHELFKEYGEVKSCRVMTDSTGVSRGFGF
VSFATPDQANAALREMNGRMLNGKPLIVNIAQRRDQRYTMLRMQFQQRLQVMMRQIQHRGPPFEGNPAR
PARNGRGGRSWQQPQPPQPPQQIQQQIQQQMQQAMPHAQARPGAGVPMPPPHPPTPSATGFSNPTPRLS
PGQAPDTPPLQPITPAELSAMSPEEKRAALGDRLFMKVYEVSPEFAPKITGMFLEMKPMDAYELLVDQK
RFTDHVTEALCVLKAHNCA

>Pser2
MTFSGPNPSIWVGGLDLSLNEQKLYDYFVRIGPVASVRVCVDSATQKSLGYGYVNFQDPADAEKALDLV
GSKLGNRYLRIAKIQRDPAKRRSGASNIVVKKLPATIDTYALKEMFSKFGRLTSIVLACDEKGVSRGYA
RISFEREESAVEAVKEMNGTEVDGQPILVERYQPQHHEELIKQFANLYVKNLNPSVTDDQLREFFSKYG
NVTSAKVRDLGEIKTDVGFGYVAFGEHDDAVKAVEALNGKECEISQKGTALDVSRFRSRGERMRDLERQ
RHERAKQYMNYPNLYVKGFDETVTSERLKELFESYGETISVRVMMDSETGLSHCFGFVSMKDHAAAAQ
AIQMLNGSTFLSSRPLFVTYALRKDVRRQNLEERSKQQFRIRQNHMGGGGAGGMGGPPSIGFMGGGPKI
FGNMSMPFMNPRMPMMPMAAAAGGFGMGRPMGGGPAPMNQMRVARPMGAQKPPMHAMVPQHYAQQAPPS
QSLSAMLPNLTPDQQRNVLGERLYSYIVRSHPTLAAKVTGMLLEMDMTEIVNMLENPTLLDNKISEALD
VLNHHMGV

>Pser3
MSFVPSQRTSLYVGDIPLDLPYPEDTLSSLFNSVAPVISLRVCRDITTQKSLGYAYINFSNTADAEKAL
NVLNYTDIIPGRQIRIMFSMRDPMIRKSALNNVCITNLSPSINAKALHKLFSEFGSILSCKVALDKDGN
SKGYGFVQFETPEGAKSALQMSGRRIGDMEVNVVPYMRKSERDAQREKTFTNVYANNILKTATEEDVKK
VFEKFGKVTSLFLSGEPKHNTKFALANFESHDDAVKAIEGLNNSTECEISDPEQPLMVCRALKKSERAR
VKPKFPAIYQNQGRNLYVKHLADDITQEELENLFAPYGQIESC

>Egra_a
MATQQPQQPNASLYVGDLPAEITEAMLFDLFKAVGPVLSIRVCRDAVTRRSLSYAYVNFQNPVDAERAL
ETLNYHQIKGSPIRIMWSHRDPSLRKTGSGNIFIKNLHKSIDNQTLYDTFSQFGNILSCKVATDPNGES
RGYGFVHFESEESAKAAIEKVNGMLLKSMQVFVGPFVRRALRIQEHAGSYTNVYIKQVKTGITEEEISG
FFGKIGPIESHVLKHDRKDRPFAFVDFKNHDDAVKAVTEFHDKEIEGITEEGQKIYIGRAQKKGERLEE

QRKRVSQLRARKMHEYSGSNLYVKNLDDNVDDAGLKAAFEKFGEITSAKVMMDDVNPGLSKGFGFVCFK
DAESANKAISEMNGHLLGVKPLYVNVAQRKEVRRATLEVQYAARMTRMPGPSVPSAGGQPGMGYPPMYP
PTPGYSAFPPSGSYFYPPGPAAPQRAAMTQAPKWAQQQQGIPGMPFPGQMSLQQAQMPMRPPPQQQAGL
ARRVMPGQGRAPAPPQMGRGPLAGKGLPRGIPPQARPLAPPSALPPPGLRGPGVDDTLSAAALLDMAPD
QQKNALGERLFARISTSQPEYAAKITGMLLEMDVTECLNLLESPEVLDSKIAEALAVLQAHDSPDQD

>Egra_b
MSLTQNASLYVGDLPSDITEAVLFDLFKAVGPVLSIRVCRDIVTRRSLGYAYVNFQNPADAERALETLN
YHVIKGQPIRIMWSHRDPSLRKLGIGNVFIKNLHPSIDNQTLYDTFSQFGNILSCKVAVGSNGESRGYG
FVHFESEESSRAAIEKVNGMLLKSLQVYVGPFVKRSIRTHQNAQTYTNVYIKNLKKGITEEQFSEFFGK
FGEIKSKALRQDREGRPFGFADFGAHEQAAKAVEECHGKFIEEVTQEEQTLYVQRFQKKSERLDEMQKR
ARLQRARKMHEYSGSNLYVKNLGEVINDESLKEAFAKFGKITSAKVMMTDGTPSLSKGFGFVCFEDSEA
ANKAISEMNGYMLGEKPLYVNVAQRKEVRQATLEVQYASRMSRLAGQVPAQQQAGMGYPPMYGPPPGAF
YPPAGPSMYMYAGQPGRPGMIPGAAPKWGAQPMQGIPGMMAQQMLPGQPPMMGRLQQGIPPGKGRPTPG
KGLPVPGRGPPLGLGKGVVKGQPLPGPQPSLAFTRQARNKDGGAGVAGVVDQPLTSSELAGMAPEQQKN
ALGERLFARISETQPEYAAKITGMLLEMDVTETLNLLESPDVLTEKINEALTVLKAHEGSLTE

>Egra_c
MATTRVPPPVAGQTALYVGDLNHDVTEAMLFEEFKQAGPIVSIRVCRDSATRRSLGYAYVNFQSSTDAE
TCLETLNYTSFKGRPCRIMWSQRNPTLRKSNKGNIFIKSLHPQIDHKALYDTFSAFGPVLSCKVVLKPN
GESAGYGFVHFDSDEAARLAVEKVNGMSLNGKIVFVGFFERRANRLELHKQKFTNIYIKHLKSEVTKEK
LDEVFSKFGAIRSSLVATKDDGSSKGFAFVNYEEHDSAVKAIDELHDSSSEQNFDLSLPSKTLYVTRHQ
RLSERQHLREQWAKERQQRLAKYVNLYVKNLDDNIDDAKLKEFFEPFGTIISAKVMRDEQSGASRGFGF
VSFRDSEAANKATAEMNNKLGISSKPLYVGPAQKKEERRLQLEMQFAQRTKAGLFTGPALSSAPLTATA
QAFNPTGYLPTASPALPLGQAHASFLQQMQGQRPSGPRWSTEQALGRMPQAMGPPRGRVPMMGPAASAY
AAQARFQMQSRPPPKSTPYPAQPLFGGRPFTTAPHMVPPQQARAPVPAMRSTEDPTLTNEYLAGLPVDQ
QKNLLGERLFSYIDRDYPDLAAKITGMLLEMEPREILGLLGQPELLGSKVSEALEVLKRHPEEMQAAKN
LE

>Tbru1
MTIAAQGVPSGPSATKPLQVASLYVGDLDPAINEPQLVDIFKPYGTILNVRVCRDIITQRSLGYGYVNY
DDVNSASKAMEELNFKRVGEKCIRIMWQQRDPALRYSGSGNIFVKNLKEEVDSRELSLIFKKFGEILSC
KVMDDESGKSRGYGFVHFKDDDAAKAAIEKMNGDKEHADADKAALYVANFIRRNARLAALVANFTNVYI
KQLLPTVDKDVIEKFFSKFGGITSAAICKDKNGRAFAFCNFEKHDDAVKAIEASHDQEVEGVTQPGEKL
YVQRAQPRSERLIALRQKYMQCQSLGNNLYVRNFDAEFTEKDLNELFKEYGVIRSCRVMTDANGISRGF
GFVSFENADQANAALREMNGRMLNGKPLVVNIAQRRDQRLTMLKLQLQQRLQMMMHHMHPPPFGMPGHP
LQRRNARSGGRGNRPHPRQQQPQPQPEPQPPLATTVPPAAAHSVMFTAPSMGFSAVPRTPQASPAIAPD
TPPLPPISAEDLQQMSVDEQRAALGDRLYIKVHELAPDHAPKITGMFLEMNPKEALALLSNPKLMHEKV
TEALCVLKVHASSA

>Tbru2
MAAFAAASPSIWVGGLDPNLNEQKLYDHFVRVGPVASVRVCVDSVTQKSLGYGYVNFQNPADAEKALDQ
AGVKLGTKHIRIAKIQRDPSKRRSGVTNIIVKKLPPTVDTYALKEMFSKYGRLTAIGLATDEKGESRGY
ARISYEKEESAVDAVRELNGVSIDDCAITVERYQPHHREEQLKQYTNLYVKNLDPSVDDEKLKEVFSPF
GEVTSAKVRDLAGRPTVGFGYVAYATHEAAAKAVEELDDKESPLAKEGMKLSVCRFRSREERKRERERL
RRERQQQHSKYPNLYVKNFDDTVTSERLKALFDPFGETVSVSVMMDKATKVSRCFGFVSFKEQSSAAQA
IQELHGSTALGPRPLFVSYALRKDARRQTLEDMRNKQPRMRQPPMGGLMGGMMGPQLSFMNPPAMFNGM
HFMNTRMPMMPSTMGMGGPMRPMGPTPMNQVRARPGPQRPPMQSMMAPQQQSHPQIPQPPVAQGQNLST
VLASMTPDQQKNVLGERLYNYIVRNNPSFAAKVTGMLLEMDNSEILNLLDNHSLLDTKVQEALDVLNRH
IGM

>Lmaj2
MAFTGPNPSIWVGGLDPDLQEQKLYDYFVRIGPVTSVRVCVDSATQKSLGYGYVNFQDPADAEKALDQA
GSKLGSRYLRIAKIQRDPSKRRSGVNNILVKKLPKSVDTYALKEMFSKFGRLTAIGLACDEKGESRGYA
RISFEREESAVDAVREMDGMEMDGQAIVVERYQAQHRDELLKQFTNLYVKNLDPAVTDEKLRAFFAKYG
EVSSAKVRDLGAAQSEAGLGYVAFQKHENAARAVEELNGKECEIAKAGSPLDVSRFRSREERQRDRERQ
RRERAQQHSKYPNLYVKGFDDTVTSERLEELFQRYGETVSVTVMMDKETGVSRCFGFVSMKDQNAASQA
IQELNGSTFLSPRPLFVTYALRKDARRQNLEERSKQFRVRQNPMGGPGMGAMPPIGFMGPQMFNNVNMP
FMNPRVPIMPMNGMNGIGGMNGMGGMNGVGGVGGMGGMGGMGGMGGMGGMGGMGSMGGMGGMARPM
APNAMSQMRSRPMPQKPPMQSLMPQQHQQAPPQGQNLAAVLANLNPEQQKNVLGERLYSYIVRSHPSVA
AKITGMLLEMDNAEILNMLDSPTMLDSKIAEAQDVLNRHMSV

>Lmaj3
MVAPAQRTSVYVGDLPIDLPRPEEAINNLFSTVAPVVSVKVCRDMATQRSLGYGYVNFQTTADAEKVID
ALNYTGIAPGRQIRVMFSIRDPLQRKSGMNNVFVKKLDTAINAKELQAAFSKCGRVLSCKVALDSAGNS
KGYGFVQFETADGTKAALEMNGSKLGDSEVVVAPFVRRVDREAMAAKSFRNIYIKNITASATEADVKAI
VEEFGKVDSLFLSEHARFPTKFALVAFEEHQAAVQAIAALNESEESGLTEKAVKLVVCRALSKSERDRE
KKKAASLYQNHGRNLYIKHLPDDITDDRLREIFEPFGKITSCAIMKEPNGTLKGFAFVCFEDKQHASAA
LRSLNGHPLEHSAKPLYVSHAEQKDMRIRLLQQRRAAMRHQSRMAPLMNTFPQQWPRHPFPHMVPPMMP
PPPPPNMGMPQFMSGPMIRRPVMEPHLMQGEPMRPPNRYTQAREQYPPQQRQDGVDMNYLSTLSPEQQK
NYLGELLYSRILPLESSNAAKITGMLLEMSREEIFEILADHFALLSKIQEANAVLQQHTGN

>Lmaj1
MAAAVQEAAAPVAHQPQMDKPIEIASIYVGDLDATINEPQLVELFKPFGTILNVRVCRDIITQRSLGYG
YVNFDNHDSAEKAIESMNFKRVGDKCVRLMWQQRDPALRYSGNGNVFVKNLEKDVDSKSLHDIFTKFGS
ILSCKVMQDEEGKSRGYGFVHFKDETSAKDAIVKMNGAADHASEDKKALYVANFIRRNARLAALVANFT
NVYIKQVLPTVNKDVIEKFFAKFGGITSAAACKDKSGRVFAFCNFEKHDDAVKAVEAMHDHHIDGITAP
GEKLYVQRAQPRSERLIALRQKYMQHQALGNNLYVRNFDPEFTGADLLELFKEYGEVKSCRVMVSESGV
SRGFGFVSFSNADEANAALREMNGRMLNGKPLIVNIAQRRDQRYTIVRLQFQQRLQMMMRQMHQPMPFV
GSQGRPMRGRGGRQQLGGRAQGHPMPMPSPQQPQGAAQPQGFATPSAVGFVQATPKHSPGDVPETPPLP
PITPQELESMSPQEQRAALGDRLFLKVYEIPPDVAPKITGMFLEMKPKEAYELLNDQKRLEERVTEALC
VLKAHQTA

>Lbra3
MAAPVQRTSVFVGDLPVDLPRPEEAINNLFSSIAPVVSVKVCRDIATQRSLGYGYVNFQTTADAEKVID
ALNFTGIAPGRYIRVMFAIRDPLQRKSGANNIFVKKLDAAVSAKALQAAFSRCGRVLSCKVALDSEGHS
KGYGFVQFETADGAKAALDMNGAKVGDSEVEVAPFVRRVDREAMAAKSFRNIYIKNIKATATEVDMRTT
LEKFGKVTSLFLAEHAPFLTKFALAVFEEHEAAVKAIAELNESEESGLTEEAVKLVVCRALSKSERDRE
RKKTASLYQNHGRNLYVKHLPDDITDDKLREIFAPFGKITSCAIMRESNGSLRGFAFVCFEDKQHATAA
MRELNGRSLESSKKPLYVSQAEQKDMRIRLLQQRRAAMRHQTRMAPPMNTFPQQWPRQPFPHMVQPMML
PPPPPNMGMPQFMSGPMMRRPVMDAHPRQGEPMRPPNRYTYPREQYQPQPQPQPQPQPRQDGIDVNYLN
TLSPEQQKNYLGELLYSRIMPVESFNAAKITGMLLEMSREEIFEVLRDHFALLAKIQEANAVLQQHSGN

>Lbra2
MAFSGPNPSIWVGGLDPDLQEQRLYDYFVRIGPVTSVRVCVDSATQKSLGYGYVNFQDPADAEKALDQA
GTKLGSRYLRIAKIQRDPSKRRSGVNNILVKKLPKTVDTYALKELFSKFGRLTAIGLACDEKGESRGYA
RISFEREESAVEAVKEMDGMEMDGQAIVVERYQAQHRDELLKQFTNLYVKNLDPAVTDEKLRAFFARYG
AVSSAKVRDLGGVQSEVGLGYVAFEKHEDAARAVEELNGKECEIAKAESTLDVSRFRSREERQRDRERQ
RRERAQQHSKYPNLYVKGFDDTVTSERLEELFQRYGETVSVTVMMDKETGMSRCFGFVSMKDQNAASQA
IQELNGSTFLCPRPLFVTYALRKDARRQNLEERSKQFRVRQNPMGGPGMGGMPPVGFMGPQMFNSVNMP
FMSPRVPIMPMNGMNGIGGMNGMGGMNGMNGMGGMGGMGGMGTMGGMGGMGRPMAPNAMSQMRSRPMPQ
KPPMQSLMPQQHQQPPPQGQNLAAVLANLNPEQQKNVLGERLYSYIVRSHPSVAAKITGMLLEMDNSEI
LSMLDSPVMLDSKIAEAQDVLNRHMSV

>Lbra1
MTTTVQETAAPVAQQPQVSKPMQIASIYVGDLDAAINEPQLVELFKPFGTILNVRVCRDIITQRSLGYG
YVNFDNHHSAERAIESMNFRRVGDKCVRLMWQQRDPSLRYSGNGNVFVKNLEKDVDSKSLHDIFTKFGS
ILSCKVMEDEEGKSRGYGFVHFKDEISAKDAIVKMNGAADHASEDKKALYVANFIRRNARLAALVANFT
NVYIKQVLPTVNKEVIENFFAKFGGITSAAACKDKSGRVFAFCNFEKHDDAVKAVEAMHDHHIDGITAP
GEKLYVQRAQPRSERLIALRQKYMQHQSLGNNLYVRNFDPEFTGADLLELFKEYGDVKSCRVMMSESGA
SRGFGFVSFSNADEANAALREMNGRMLNGKPLIVNIAQRRDQRYTMLRLQFQQRLQMMMRQMHQPMPFV
GGQGRPMRGRGGRQQQGGRAQGHPMPMPSPQQSQGFATPSAVGFVQATPKHSPGQVPETPPLPPITPQE
LESMSPQEQRAALGDRLFLKVYEIAPELAPKITGMFLEMNPKEAYELLNDQKRLEERVTEALCVLKAHQ
TV

>Linf3
MVVPVQRTSVYVGDLPIDLPRPEEAINNLFSTVAPVVSVKVCRDMATQRSLGYGYVNFQTTADAEKVID
ALNYTGIAPGLQIRVMFSIRDPLQRKSGMNNVFVKKLDTAINAKELQAAFTKCGRVLSCKVALDSAGNS
KGYGFVQFETAEGAKAALDMNGSKLGDSEVVVAPFVRRVDREVMAAKSFRNIYIKNIAAAATEADVKAA
AEKFGKVNSLFLSEHAPFPTKFALVAFEEHEAAVQAIAALNESEESGLTEKAAKLVVCRALSKSERDRE
KKKAASLYQNHGRNLYVKHLPDDITDDKLREIFEPFGKITSCAIMKEPNGTFKGFAFVCFEDRQHASAA
LRSLNGQPLEHSKKPLYVSHAEQKDMRIRLLQQRRAAMRHQSRMVPPMNTFPQQWPRHPFPHMVPPMMP
PPPPPNMGMPQFMSGPMIRRPAMEPHLMQGEPMRPPNRYTQPREQYPPQQRQDGVDMNYLSTLSPEQQK
NYLGELLYSRILPLESSNAAKITGMLLEMSREEIFEILADHFALLSKIQEANAVLQQ
HTGN

>Linf2
MAFTGPNPSIWVGGLDPDLQEQKLYDYFVRIGPVTSVRVCVDSATQKSLGYGYVNFQDPADAEKALDQA
GSKLGSRYLRIAKIQRDPSKRRSGVNNILVKKLPKSVDTYALKEMFSKFGRLTAIGLACDEKGESRGYA
RISFEREESAVDAVREMDGMEMDGQAIVVERYQAQHRDELLKQFTNLYVKNLDPAVTDEKLRAFFAKYG
EVSSAKVRDLGAVQSEAGLGYVAFQKHEDAARAVEELNGKECEISKAGSPLDVSRFRSREERQRDRERQ
RRERAQQHSKYPNLYVKGFDDTVTSERLEELFQRYGETVSVTVMMDKETGVSRCFGFVSMKDQNAASQA
IQELNGSTFLSPRPLFVTYALRKDARRQNLEERSKQFRVRQNPMGGPGMGAMPPIGFMGPQMFNNVNMP
FMNPRVPIMPMNGMNGIGGMNGMGGMNGVGGVGGMGGMGGMGGMGGMGGMGGMARPMAPNAMSQMR
SRPMPQKPPMQSLMPQQHQQAPPQGQNLAAVLANLNPEQQKNVLGERLYSYIVRSHPSVAAKITGMLLE
MDNSEILNMLDSPTMLDSKIAEAQDVLNRHMSV

>Linf1
MAAAVQEAAAPVAHQPQMDKPMQIASIYVGDLDAAINEPQLVELFKPFGTILNVRVCRDIITQRSLGYG
YVNFDNHDSAEKAIESMNFKRVGDKCVRLMWQQRDPALRYSGNGNVFVKNLEKDVDSKSLHDIFTKFGS
ILSCKVMQDEEGKSRGYGFVHFKDETSAKDAIVKMNGAADHASEDKKALYVANFIRRNARLAALVANFT
NVYIKQVLPTVNKEVIEKFFAKFGGITSAAACKDKSGRVFAFCNFEKHDDAVKAVEAMHDHHIDGITAP
GEKLYVQRAQPRSERLIALRQKYMQHQSLGNNLYVRNFDPEFTGADLLELFKEYGEVKSCRVMVSESGA
SRGFGFVSFSNADEANAALREMNGRMLNGKPLIVNIAQRRDQRYTMLRLQFQQRLQMMMRQMHQPMPFV
GGQGRPMRGRGGRQQLGGRAQGHPMPMPSPQQPQAPAQPQGFATPSAVGFVQATPKHSPGDVPETPPLP
PITPQELESMSPQEQRAALGDRLFLKVYEIAPELAPKITGMFLEMKPKEAYELLNDQKRLEERVTEALC
VLKAHQTA

>Tcon1
MTVVPQGAPPAAPAAKQLQVASLYVGDLDPAISEPQLVEIFRPYGTILNVRVCRDIITQRSLGYGYVNY
DNANSATKAMEEMNFKRVGEKCIRIMWQQRDPALRYSGNGNVFVKNLKGEVDSRELSLIFKKFGEILSC
KVMDDESGNSRGYGFVHFKDDNAAKSAIESMNGVTEYADEKKTALYVANFIRRNARLAALVANFTNVYI
KQILPTVDKAIIEKFFSKFGGITSAAICKDKNGRAFAFCNFEKHDDAVKAIEEFHDHEVEGVTQPGEKL
YVQRAQPRSERLIALRQKYMQCQSLGNNLYVRNFDPEFTEKDLNELFKEYGVIRSCRVMTDANGVSRGF
GFVSFENADQANAALREMSGRMLNGKPLVVNIAQRRDQRFTMLRLQLQQRLQMMMRQIHPPPFGMPRQM
PQRRRGRQHPRGHDQMPSLPVMPPVIPRNDMFNTPSMGVSAAPRTPQASPATAPDTPPLPPITAEDLQN
MTMEEQRAALGDRLYIKVYELAPDHAPKITGMFLEMNPKEAHTLLTNQRLLQDKVTEALCVLKVHASNS

>Tcon2
MSVFPTASPSIWVGGLDPNLNEQKLYDHFVRVGPVASVRVCVDTVTQKSLGYGYVNFQNPADAEKALDQ
AGVKLGTKHIRIAKIQRDPSKRRSGVTNIVVKKLPSTVDTYALKEMFSKFGRLTAIGLATDEKGESRGY
ARISYEKEESAVEAVRELNGVSIDDCSITVERYQPHHREEQLKQYTNLYVKNLDPSVDDEKLKEVFAKF
GEVTSAKVRDLGNNSSVGFAYVAYATHEAAAKAVEELDEKSSTLAKEGMKLSVCRFRSREERQRERERV
RRERQQQHGKYPNLYVKNFDDTVTSEKLKELFERFGETVSVSVMMDKATQVSRCFGFVSFKEQSAASQA
IQELHGSTALGPRPLFVTYALRKDARRQTLEDIRNKQPRMRQPPMGGLMSGMMAPQLGFMNPPTMFNGL
PFMNPRMPMMPSAMGMGGPMRPMAPAPMNQVRARPAPQRPPMQAMMAPQQQPQLHPQQPQPPVPQGHNL
STMLASLPPEQQKNVLGERLYNYIVRNNPSVAAKVTGMLLEMDNSEILNLLDSPSMLDTKVQEALDVLN
NHLRA

>Tcru1
MLAVNKPMAATQEEITPTVPVTTKALQVASLYVGDLDPVVTEPHLVELFKPFGTILNVRVCRDIITQRS
LGYGYVNFNSHDSAAKAMEALNFKRVGDKCMRIMWQQRDPTLRYSGNGNIFVKNLKNEVDSRELSVIFK
KFGDILSCKVMEDEEGKSRGYGFVHFKNDNAAKEAIENMNGEKDHADEEKKMGLYVANFIRRNARLATL
VANFTNVYIKQVLPTVDKEVIEKFFSKFGGITSSATCKDKNGRVFAFCNFEKHEDAVKAIEASHEQFVD
GVVPPGEKLYVQRAQPRSERLIALRQKYMQCQTLGNNLYVRNFDPEFTEENLHELFKEYGVIRSCRVMT
DANGNSRGFGFVSFENADQANAALREMNGRMLNGKPLIVNIAQRRDQRFMMLRLQFQQRLQAMMRRMHS
MPFVSHGHLPQRRNARGTQRGGGGGGGVGGGGAGRIQAPPPMPPPVQQDMFATPSMAFAPPRTPQPSPG
VAPDTPPLPPITAEDLRSMSVDEQRAALGDRLYIKVFEIAPDHAPKITGMFLEMDLKEAFTLLTNQRLL
QEKVIEALCVLKAHESTA

>Tcru2
MSNFPAASPSIWVGGLDPNLNEQKLYDHFVRLGPVASVRVCVDSVTQKSLGYGYVNFQNPADAEKALDQ
AGVKLGSKHIRIAKIQRDPSKRRSGVTNIVVKKLPPSVDTYALKEMFSKYGRLTAIGLATNENGESRGY
ARISYEKEESAIQAVQEVNGMVIDDCAITVERYQPHHREEQLKQFTNLYVKNLDPSVNDEKLKEVFSAF
GEVTSAKVRDLGANQTVGFAYVAYATHEAAAKAVEELDEKESPLAKEGMKLSVCRFRSRDERQRERERL
RRERQQQHSKYPNLYVKNFDDTVTSERLKELFERCGETVSVSVMMDRATRVSRCFGFVSFKEQSAASRA
IQELHGSTALGPRPLFVTYALRKDARRQTLEDMRNKQPRMRQPPMGSLMGGMMGPQLGFMGPQAMFNGV

PFVNPRMSMMPTPMGMGGQLRPMGPTPMNQVRARPMPQRPPMQPIMAPPPQPQSLASQGQNLSTVLANL
TPEQQKNVLGERLYNHIVAINPAAAAKVTGMLLEMDNGEILNLLDTPGLLDAKVQEALEVLNRHMNV

>Tva1
MDPNWKEVFVGDLPGSVDENFIKEIFKDYGSFPSGTVTVKKHKSLDKSFAFVTFESHELAKRAISEVNY
TKLDGVPIRILWSDPGTKRAIKNNVGALFIRGLDENIEVSQLHDAFSNFGEIVSCKIPLTNGKSRGYGF
ITFYKEDDAKRAKTDLADASINGKPIQIEFYQKPTRKNPEETFTNVFIKPLPADIFKTDDDLANFFKEF
GDFVVTGKANPAIKRKEDGSSCEFGFCNFKHHEDAVKAVDALNGKQHESGKVTFSCCRAQTKAERQAFL
AKQSAEFRRRLNEETRGRNLYVKNFDQSVTDEQFKEYFEQFGEVEKCSIRREAQEPHESKGFGFVLFKT
KESAQNALENAVITPLNGKTPYIGLFKMKEEREREKASNQRKQQPKGAMIPNTIGGPVIPTQQIMMQSM
MMPQVPVSAPATSKDLLKNELTERNISGSKLKAILSSISEEQAKSLCSDQEKLNHWVEEKTKM

>Tva2
MATLTTQIFVGDLPKDIDESFLKQFFAESGEIIPNSGIVLKKHKTLDSSFAFVTFATHEQAAKAMKNLN
YTKLDGRPIRILWCDTETKAIIKSGRGSLFINGLDESIEVSQLHDAFSNFGEIISCKIPLTDGKSRGYG
YIQFRNPENAEKAKLELADASINGKPIKIDNYNRKSRRNPDEDFTNVYIKPLPVDQVKTDEDLRKIFEP
FGEIQNPSLKKDENGNSKGFGFCNFKLHEDAVKAVEGLNGQEMFGVTLQVNRLMSKREKELYNIKKHTE
KVEKFAEETNGRNLYVKSFSKDVTDKEFQEYFSKFGEIELFKIERVPETKESKGFGFVLYKSKEDAQNA
IEMAMLERLHGDLPYVGFFQTKAAHERVKVKNTHPRPSAVPAIAGNLMENSMTTIARQYQPVEDNSPKG
QLKQLLQEKGVLGTNLKRLLSSVSAEQAVNLVKEQSKFEIWYSHNAN

>Tva3
MDLNWKELFVGDLPGYVDGNFIKELFREYGNFPSGTITVKKHKSLDKSFAFVTFESHELARRALLEVNY
TKLDGVPIRILWSDPATKRAIKKDFCTLIIFDLDEYIEAAQLHDIFSNFGEVVSCKIPLTNGKPRGDGY
VTFYKEEDAMRVKNDLAQASINGKPIQIVLYCKPTRKNSEETFTNVFIKPLLVKYFKTNEDLAKLMKDF
GEFVNPSIKFNDDGTSSEFGFCNFMYHQDAVKAVESLNGKMHISGEFEFVCCRAQTKAERQAFLAEQSA
EFRRRLYEETRGRNLYIKNIDRSINDEEFEEYFKQFGEVEKCLISREAQEPHESKGFGFVLFKTKEGAQ
NALKNTIITPLKGKILYVNYFIMKEEREHILPMDQPKTYLNFIKTKLSERGFPNKQKKKNSLFNLRRTS
SRTLQ
