# Supplementary material for: Differential Localization of the Two T. brucei Poly(A) Binding Proteins to the Nucleus and RNP Granules Suggests Binding to Distinct mRNA Pools
Source: PLoS One. 2013 Jan 30;8(1):e54004. doi: 10.1371/journal.pone.0054004 (PMC3559699; doi:10.1371/journal.pone.0054004)
Supplement: Table S2 — Plasmids used in this work. Some of the plasmids have been previously described [63] . (PDF) [file pone.0054004.s011.pdf]

Table S2: Plasmids used in this work. Some of the plasmids have been previously described (Kramer et al 2012).

	Gene ID	nucleotides of ORF used for targeting / RNAi / expression	restriction enzyme used for linearization	backbone plasmid	backbone plasmid described	expression vector	selectable marker
PABP1-4Ty1	Tb09.211.0930	592-end	BsgI	p3074		p3074	NEO
PABP1-eYFP	Tb09.211.0930	123- end	NsiI	p2710 with HYG	Kelly 2007	p2949	HYG
PABP2-mChFP	Tb09.211.2150	537-end	XcmI	p2705	Kelly 2007	p3279	NEO
PABP2-eYFP	Tb09.211.2150	539- end	XcmI	p2710	Kelly 2007	p3295	NEO
mChFP-DHH1	Tb927.10.3990	1-466	NheI	p2679 with BSD	Kelly 2007	p2845	BSD
eIF4E1-eYFP	Tb11.18.0004	162- end	NruI	p2710	Kelly 2007	p3519	NEO
eIF4E1-mChFP	Tb11.18.0004	162-end	AarI	p3086	n.d.	SK85	HYG
eIF4E4-eYFP	Tb927.6.1870	361- end	BspEI	p2710	Kelly 2007	p3522	NEO
eIF4G3-eYFP	Tb927.8.4820	1319- end	BbsI	p2710	Kelly 2007	p3549	NEO
PABP1-mChFP	Tb09.211.0930	123-end	NsiI			p3071	HYG
PABP1 ALRQKY::RLRRER	Tb09.211.0930	full length	NotI	p2216	Kelly 2007	p3404	PHLEO
PABP2 RLRRER::ALRQKY	Tb09.211.2150	full length	NotI	p2216	Kelly 2007	p3405	PHLEO
GGHESSVE	random sequence		NotI	p4186	Sunter 2011	p4255	BSD
RGHKRSRE	NLS of LA protein		NotI	p4186	Sunter 2011	p4246	BSD
RLRRER	predicted NLS of TbPABP2		NotI	p4186	Sunter 2011	p4190	BSD
RFRSREERKRERERLRRERQQQHSKYP	predicted NLS of TbPABP2		NotI	p4186	Sunter 2011	p4187	BSD
RSREERKRERERLRRER	predicted NLS of TbPABP2		NotI	p4186	Sunter 2011	p4188	BSD
RERERLRRERQQQHSKYP	predicted NLS of TbPABP2		NotI	p4186	Sunter 2011	p4189	BSD
PABP1-NLS of LA protein-eYFP	Tb09.211.0930	123- end	NsiI	p2949	Kramer 2008	p3633	HYG
PABP1 RNAi	Tb09.211.0930	full length	NotI	p2T7-177	Wickstead 2002	p2319	PHLEO
PABP2 RNAi	Tb09.211.2150	full length	NotI	p2T7-177	Wickstead 2002	p2287	PHLEO

Kramer S, Marnef A, Standart N, Carrington M (2012) Inhibition of mRNA maturation in trypanosomes causes the formation of novel foci at the nuclear periphery containing cytoplasmic regulators of mRNA fate. J Cell Sci.
